# Impact of antidote quantity, timing and prehospital strategies in nerve agent mass casualty events: a simulation study

**DOI:** 10.3389/fpubh.2025.1640554

**Published:** 2025-08-26

**Authors:** Ruben De Rouck, Mehdi Benhassine, Michel Debacker, Filip Van Utterbeeck, Jan Vaes, Isabell Meskens, Ives Hubloue

**Affiliations:** ^1^Research Group on Emergency and Disaster Medicine, Vrije Universiteit Brussel, Brussels, Belgium; ^2^Simulation, Modelling, and Analysis of Complex Systems, Department of Mathematics, Royal Military Academy, Brussels, Belgium; ^3^Medical Component of Belgian Armed Forces, Neder-over-Heembeek, Belgium

**Keywords:** mass casualty incidents, computer simulation, nerve agents, sarin, medical countermeasures, CBRN

## Abstract

**Introduction:**

Mass casualty incidents (MCIs) involving nerve agents pose major challenges for emergency medical response due to rapid symptom onset, hazardous environments, and operational uncertainties. Several gaps remain in the knowledge about the prehospital response to nerve agent MCI treatment strategies and logistical decision-making. To address these gaps, this study uses Discrete Event Simulation to evaluate the impact of advanced medical stabilization (AMS) team arrival time, antidote availability, and evacuation policy on patient survival during an urban chemical-traumatic MCI with a subway sarin release scenario.

**Methods:**

A validated simulation model (SIMEDIS) was adapted to represent the full prehospital response chain, including triage, antidote administration, AMS, dry decontamination, further on-site stabilization in the forward medical post and transport to categorized hospitals. Two transport policies were modeled: Scoop&Run (rapid transport of victims to hospitals) and Stay&Play (on-site stabilization before transport). We simulated various AMS team arrival times and antidote availability scenarios to assess their impact on survival. Locations of deaths were analyzed to identify critical points of failure in the medical response chain.

**Results:**

AMS team arrival time, antidote availability, and evacuation policy significantly influenced mortality among the 25 salvageable victims. The number of deaths ranged from 8.0 (32%) in the most favorable case to 23.8 (95.2%) in the least favorable. Earlier AMS team arrival and greater antidote availability were associated with fewer deaths, particularly under the Scoop&Run policy. Stay&Play resulted in more deaths unless medical and transport capacity were significantly enhanced. Location-of-death analysis revealed preventable bottlenecks, especially during decontamination and hospital transport under the Stay&Play model.

**Discussion:**

The results highlight the importance of rapid hospital transport, swift antidote availability and administration during urban chemical MCIs. AMS team arrival time emerged as the strongest predictor of preventable mortality, showing a sigmoid-shaped curve where delays beyond 11 min led to sharp increases in death. Antidote supply showed a dose-dependent effect, but the impact diminishes with delayed administration, underscoring the need for timely delivery over sheer volume. To reduce preventable deaths in chemical MCIs, policy makers should focus on streamlining AMS team deployment, prioritizing rapid evacuation, and addressing logistical bottlenecks in decontamination and transport.

## 1 Introduction

The medical response to mass casualty incidents (MCI) involving chemical warfare agents is complex and requires specialized equipment, knowledge and skills (1). Among these chemical warfare agents, nerve agents are particularly complex due to the hazardous environment and the rapid onset of life-threatening symptoms that demand immediate intervention (2). Currently, several critical knowledge gaps remain regarding the treatment and medical extraction of exposed casualties—particularly in the context of nerve agent attacks. These gaps may contribute to preventable prehospital morbidity and mortality, highlighting the urgent need for evidence-based protocols and operational strategies (3).

Recognizing these deficiencies, policymakers have prioritized national-level planning and structural preparedness to improve chemical MCI response capacity. For example in Belgium, due to the increased awareness of national vulnerability the Federal Public Service for Health commissioned the Belgian Health Care Knowledge Center (KCE) to evaluate the country’s preparedness for CBRNe (chemical, biological, radiological, or nuclear substances, potentially via explosives) incidents. To optimize medical response capacity during MCIs the KCE proposed a dynamic framework incorporating specialized advanced medical stabilization (AMS) teams as well as a revised hospital categorization system (4). While this framework provides a structure for organizing and stratifying the emergency response, it leaves many operational decisions unaddressed. Key questions include: how many victims should a local AMS team be prepared to treat, where should the AMS team be stationed, and should victims be stabilized on scene or quickly distributed across the newly categorized hospitals?

To explore these unresolved operational questions and test the proposed response strategies, we opted for computer simulation. Simulation has emerged as an operational research tool for disaster preparedness and healthcare system planning, offering a cost-effective, reproducible alternative to resource-intensive field exercises and drills (5, 6). These traditional methods, while useful, are limited by high costs, time demands, and a lack of reproducibility. In contrast, simulation allows for controlled testing of strategic decisions and resource allocations in rare, high-stakes events such as MCIs involving chemical agents. Various simulation techniques have been applied in healthcare, but Discrete Event Simulation (DES) is especially well-suited to this context. DES enables modeling of patient flow through complex medical systems, capturing stochastic factors, time-dependent interventions, and queuing dynamics with high resolution (7, 8). Unlike Agent-Based Models, which focus on individual behavior and interaction, or System Dynamics models, which analyze aggregated flows, DES provides a balance between detail and computational efficiency. It is particularly effective for identifying bottlenecks, testing “what-if” scenarios, and guiding policy-relevant operational decisions under conditions of uncertainty and urgency (9, 10).

The aim of this study is to assess how AMS team arrival time, antidote availability, and evacuation policy affect patient survival in a simulated urban mixed chemical-traumatic MCI.

## 2 Materials and methods

The Simulator is based on a previously validated DES model called SIMEDIS (Simulation for the Assessment and Optimization of Medical Disaster Management) (11, 12). This model simulates the entire chain of medical response from exposure to ED admission and has previously been used to assess resource allocation and triage effectiveness in disaster settings and military settings (12–15). This simulation model was iteratively adapted, verified and validated for the aims of this study.

To evaluate the study’s aims in a realistic context, we opted to design a scenario involving chemical weapon dispersal in a subway station based on previous risk assessment. Several chemical warfare agents were considered for the creation of the simulation scenario. The nerve agent sarin (NATO designation GB) was selected due to its high lethality, rapid action, and recent use in both military and terrorist contexts (16, 17). The amount of sarin released needs to be carefully calibrated to reflect a realistic mass casualty incident, with a victim count exceeding local emergency medical services (EMS) surge capacity and a case mix focused on clinically significant exposures. We settled on a release where approximately 650 grams of high-purity GB is released via aerosol devices 1 min before a train enters the subway station during rush hour. In total 986 people are present in the station. When victims show signs of breathing difficulties, convulsions and loss of consciousness, a panic-induced stampeding occurs resulting in victims with chemical and/or traumatic injuries.

### 2.1 Simulator process overview

The victims are injected at the disaster site at the start of the simulator. Victims without neither chemical nor traumatic injuries leave the station and are removed from the simulation. Mobile victims who are intoxicated or injured are directed to the a casualty collection point (CCP) between the two main subway entrances by members of the fire department in personal protective equipment (PPE). Victims who cannot self-evacuate due to their chemical and/or traumatic injuries are moved by Hazardous Materials (HAZMAT) teams to the CCP after being pre-triaged based on injury and/or intoxication severity.

At the CCP, a triage zone is set up in accordance with the chemical contingency plan. Triage is performed by the first four mobile medical teams (MMTs) arriving at the scene, consisting of an emergency physician and nurse donned in PPE. Victims are triaged using the SALT (Sort, Assess, Life-saving Interventions, Treatment/Transport) method, which includes the application of life-saving interventions (LSI) before determining the triage category (18). The LSI consist of antidote administration (when available), stopping life-threatening hemorrhage, airway opening - including 2 rescue breaths in unconscious children - and tension pneumothorax decompression (19). The ‘expectant’ triage category is not used, due to difficulties in accurately distinguishing survivable from non-survivable cases in a scenario with relatively low lethal exposures, and the proximity of several major hospitals.

After triage, victims are moved to the decontamination zone of the CCP waiting in the decontamination queue based on their triage priority and where they undergo dry decontamination. After their arrival in the CCP, the AMS team provides stabilizing care for the severely injured and/or intoxicated victims of the decontamination queue. Both AMS and decontamination are explained in more detail in 2.4.

Once decontaminated, T3 victims are transferred from the transport zone of the CCP to outpatient clinics in non-EMS transport means and exit the simulator. In the Scoop&Run evacuation policy, theT1 and T2 victims are transported directly from the transportation zone of the CCP to the hospital’s ED for definitive care. In the Stay&Play evacuation policy, the T1 and T2 victims are moved to a Forward Medical Post (FMP) for further stabilization, based on their triage category by a set of 5 ambulances.

**Figure 1:**
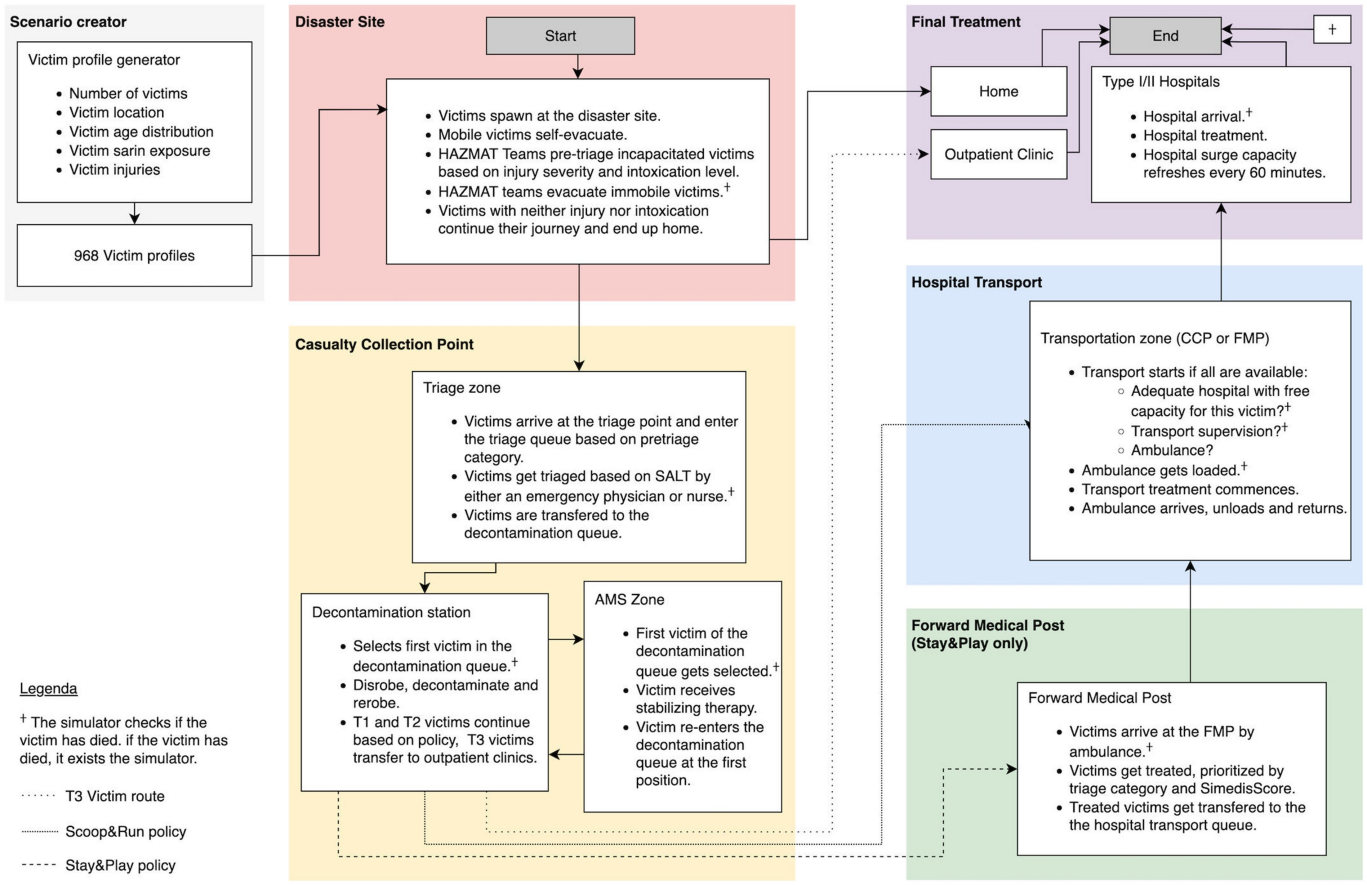
Victim flows and resources of the basic prehospital simulation model. Patient flows are marked by arrows. A full list of all resources included in the model and their specific capacity is in the STRESS-DES checklist provided as Supplementary material. Adapted from Clark et al. (2025) (48).

Finally, these victims are transferred from the transportation zone of the FMP to appropriate hospitals. Transport and treatment dynamics are explained in more detail in sections 2.5 and 2.6. A schematic representation of the simulation process is represented in Figure 1. A generalized overview of the simulation process is represented in Figure 1.

### 2.2 Victim profiles

As described in previous publications, the number of victims, the injury assignment and the distribution of injuries were determined by combining traumatic and chemical injury models, a chemical dispersion model and an evacuation model (14, 20, 21). The victim’s health state is modeled by the SimedisScore which incorporates 5 clinical parameters: Glasgow coma scale, heart rate, systolic blood pressure, respiratory rate and oxygen saturation. The traumatic victim’s SimedisScore evolution follows a Gompertz curve, and the chemically intoxicated victim’s evolution is based on one of six discrete chemical injury profiles (IP) (13). Intoxication levels range from very mild (IP1) to lethal if untreated (IP6). The injury profiles are assigned corresponding to a victim’s cumulative absorbed dose and are based on military models adapted for civilian purposes. Clinical signs and symptoms were added to these victim profiles based on expert opinion in conjunction with existing animal and human experimental data as well as pharmacokinetic/pharmacodynamic models available in the literature (22, 23).

In this simulation, there are 25 preventable deaths (16 purely chemical, 8 traumatic and 1 mixed chemical and trauma). These are likely to survive until hospital admission if promptly treated (22). Out of the 986 persons present in the subway station, 51 victims are severely intoxicated: 34 non-lethal (IP5) and 17 lethal intoxications (IP6) who are assumed to die within 15–20 min, if left untreated.

### 2.3 Antidote modeling

Antidotes are modeled as doses for the treatment of 1 severely intoxicated victim. One dose consists of 3 atropine/pralidoxime auto-injectors (containing 2.1 mg of atropine and 600 mg of pralidoxime), as well as enough atropine, pralidoxime and anticonvulsants to provide the required prehospital treatment. The number of administered auto-injectors depends on the severity of intoxication. Moderately intoxicated (IP3 and IP4) victims receive 1 auto-injector, while severely intoxicated (IP5 or IP6) victims receive 3 (24). The administration of auto-injector(s) improves the victim’s health state, decreasing the IP level by 1 in the case of IP3-5. In the case of a lethal exposure (IP6) the administration of 3 auto-injectors has an ~85% effectiveness which is modeled as a probability of success based on Rodriguez and McClellan’s pharmacokinetic-pharmacodynamic (PKPD) model (23). These IP6 victims can still survive if they receive AMS treatment which includes respiratory support and higher doses of atropine. The AMS team hands over half of their auto-injector stock to the triage team 2 min after arriving in the CCP for use in the SALT triage. When the auto-injectors arrive, one ED nurse temporarily stops triaging to administer the auto injectors to the victims who still are waiting for decontamination following the standard protocol.

The theoretical maximum of applicable auto-injectors is 517, consisting of 3 auto-injectors for the 51 severely intoxicated victims and 1 for the 364 moderately intoxicated victims.

### 2.4 Decontamination and AMS treatment

After triage in the CCP victims are transferred to the decontamination queue with a priority based on their triage and SimedisScore. Due to the number of victims and known lengthy deployment times of wet decontamination units, we implemented a dry decontamination only protocol (25, 26). This dry decontamination process is provided by the fire department, consists of individual decontamination kits with absorbent materials and replacement clothing (27). The capacity is based on real life data, and the kits are available 10 min after their arrival. The decontamination process itself is modeled as a series of tracks consisting of disrobing, padding with absorbent materials and re-robing. Each of these steps are modeled as a time delay.

The AMS is implemented as a process that temporarily removes the victim with the highest priority from the decontamination queue for advanced life support according to the (MARCHE)^2^ protocol (28). AMS treatment is modeled as a decrease in intoxication severity (if not already decreased from antidote administration), as well as an increase in survival time based on subject matter expert estimation. After decontamination, T1 and T2 victims are transported either to the FMP for further stabilization or directly transported to the hospitals from the CCP depending on the evacuation policy.

The AMS team and the decontamination workers are assumed to continuously survey the queue to prioritize deteriorating victims for decontamination and stabilization. This is modeled by a continuous function that traverses the queue and takes 30 s per victim to keep each victim’s priority score up to date. The timing of 30 s was chosen to correspond to the triage duration.

### 2.5 Transport dynamics

The KCE framework distinguishes two types of CBRN hospitals. Type I hospitals must have disposal of CBRN expertise and are designated to treat severely injured and/or intoxicated victims (T1 and T2). All other hospitals are considered type II, which are expected to manage minor (T3) injuries, act as surge capacity for T2 victims, and treat walk-in patients who bypass the emergency medical system.

Hospital assignment is based on the CBRN hospital type, as well as on the treatment needs of the victims (e.g., pediatric surgery, cardiothoracic surgery, neurosurgery, major trauma center…). Hospital surge capacity is expressed per triage level and reflects the actual surge capacity as defined in the national contingency plan. It resets every hour, starting when the first victim is transported to this hospital. Victims triaged as T1 are restricted to type I hospitals while T2 and T3 can be treated at both type I and type II hospitals. Hospitals are selected based on the “closest first” philosophy, where the closest available and eligible hospital is selected according to their surge capacity. Transport of T1 and T2 victims only commences after all requirements are fulfilled and a hospital slot is available. T1 victims require either an emergency physician or a nurse for transport supervision, while T2 victims are supervised by Emergency Medical Technicians (EMTs) only.

Victims in the T3 category exit the simulator after decontamination as they are assumed to be transported to the hospitals using non-EMS transport means such as busses, taxis or non-emergency ambulances to out-patient centers.

### 2.6 Treatment dynamics

Every intervention has a stochastically variable treatment duration based on the injury severity and the available resources at the point of treatment. Treatment effects are modeled as a either a temporary or permanent increase in the victim’s health state. The effect of AMS is modeled as a decrease in intoxication severity (if not already decreased by auto-injector administration) as well as an increase in survival time of 30 min for T1 victims and 42 min for T2 victims. In the FMP victims are treated in order of triage category and then in order of their SimedisScore, with first-in-first-out as a tie breaker. Treatment in the FMP requires both an emergency physician and nurse for T1 victims, but either one can treat T2 victims. When there are concurrent requests for a health care provider for FMP treatment, the T1 victim has priority over the T2 victim, and FMP treatment has priority over transport supervision. The FMP treatment takes between 2 and 12 min based on injury and intoxication severity and increases survival time by 56 min for T1 victims and by 78 min for T2 victims. After FMP treatment, victims are put in the transport queue with a priority score based on their triage category and their health state, from which they are selected for hospital transport. Treatment during transport to the hospital is more complicated and less exhaustive due to the ambulance context. It is assumed to have the same duration as FMP treatment but only increases survival time by 26 or 40 min for T1 and T2 victims, respectively. Treatment has an immediate effect for the victim, but healthcare providers remain unavailable for the whole treatment duration and must return to the disaster site in the ambulance before becoming available again.

### 2.7 Medical director

We have extended the previously reported version of the simulator with a medical director agent called DIR-MED. This DIR-MED (and his adjunct) arrive after a driving time of 8.5 min and are responsible for the operational medical management during an MCI. The DIR-MED is implemented as a DIR-MED function, incorporating the roles and responsibilities of the DIR-MED, his adjunct, the medical regulation and transport officer as described in the Belgian Royal Decree of 22 May 2019 (29). In the scenario, this DIR-MED function takes several decisions. It dynamically distributes MMTs between FMP treatment and ambulance transport supervision. It reassigns the triage MMTs to transport or to the FMP when the last victim has been triaged. Finally, when transport to the FMP is finished, the DIR-MED function also assigns the ambulances involved to the hospital transport.

### 2.8 Simulator settings and parameters

The simulator is built in *Julia* using the *SimJulia* 0.8.2 package and *Julia* version 1.8 (30, 31). The simulator is configured to correspond to current timelines and standards of care. Drive times and resource arrivals are calculated using Dijkstra’s algorithm and data from the *OpenStreetMap* database (32). Five simulation replications were performed for every parameter combination. Simulator parameters were chosen based on the best available data. Table 1 provides an overview of the simulator parameters and their origin. Final statistical analysis was carried out using the python package *statsmodels* (33). Graphical representations were generated using *Matplotlib* (34).

**Table 1:** Summarized overview of the simulator parameters and their origin.

Input parameter	Description	Data origin
General		
Incident Recognition time	Constant 2.5 min	Expert input
MMT total	Constant 14 MMTs	Expert input
Ambulance total	Constant 29 ambulances	Expert input
HAZMAT Team composition	Constant 2 PPE workers with a stretcher and preliminary triage tokens	Real life data
Walking speed	Normal distribution, mean 1.3 m/s, truncated at 3σ to ±20% [1.05 m/s – 1.54 m/s]	Literature review
Minimal FMP Staffing	Constant 5 nurses and 5 doctors	Expert input
FMP Readiness	Constant 15 min	Local contingency plans
Triage		
Triage MMTs	Constant 4 MMTs	Expert input
Triage duration	Normal distribution truncated at 3σ to ±25% with these means and intervals Mobile victims: 5 s [3.75–6.25 s] Incapacitated: 30 s [22.5–36 s]	Literature review (19, 49).
AMS and decontamination		
AMS team Readiness	Constant 10 min after arrival	Expert input
Decontamination Readiness	Constant 15 min after start	Expert input
Decontamination tracks	Constant 20 for mobile victims Constant 10 for incapacitated victims	Expert input
Decontamination time	Mobile victims: Normal distribution, mean 3.66 min, truncated at 3σ to ±25% [2.75–4.58 min] Incapacitated victims: Normal distribution, mean 5 min, truncated at 3σ to ±25% [3.75–6.25 min]	Published own data, Literature review (1, 26, 50, 51)
Treatment		
Traumatic Treatment duration	Triangular distribution with a 20% range with the following means: T1 victims: 15 min T2 victims: 10 min	Literature review (47, 52, 53)
Chemical treatment duration	Triangular distribution with a 20% range with the following means: T1 victims: 10 min T2 victims: 5 min	Literature review (47, 54)
AMS duration	Triangular distribution with a 20% range with the following means: T1 victims: 4 min T2 victims: 2 min	Expert input, Literature review (47, 54)
Transport		
Hospitals	All hospitals within 50 km radius	Expert input
Ambulance loading time	Normal distribution, mean 2 min, truncated at 3σ to ±20% [1.6–2.4 min]	Real life data
Ambulance unloading and handover time	Normal distribution: mean 15 min, truncated at 3σ to ±20% [12–18 min]	Real life data
Surge capacity time	Constant 60 min.	National contingency plans

The simulation study was performed in 4 experiments in total. These steps are described in accordance with the standardized STRESS guideline (35). Table 2 contains a summarized overview of the STRESS DES checklist. The full checklist is available in the Supplementary material. The first experiment is designed to assess the impact of AMS team dynamics and evacuation policies on the number of preventable deaths. The AMS team arrival time and the number of available antidotes were varied according to relevant numbers after discussions with stakeholders and experts. The experimental design parameters are summarized in Table 3. The AMS team arrival times were varied from 5 min to 25 min in 2-min intervals. The impact of the AMS team arrival time shows a time-dependent effect on the number of preventable deaths with two plateaus and a sigmoid transition between 11 and 13 min, as well as a positive effect of the number of antidotes on mortality. To further understand the impact of the number of antidotes a second simulation experiment was performed, focused on this sigmoid transition zone. We added a negative control (i.e., no antidotes), as well as an extra datapoint of 60 antidote doses. To incorporate a clear benchmark for optimal performance we also added the theoretical maximum of applicable antidotes (415 antidote doses and 517 auto-injectors). Although this is likely much higher than necessary, it provides certainty that it is an optimal solution. This experiment also acts as a sensitivity analysis for the simulator. During the analysis of the preliminary results of these experiments one subject matter expert suggested modeling a reduction from 3 to 1 auto-injector in the SALT triage of severely intoxicated victims. This experiment was conducted with the same specifications as in the first experiment, except that 10 min after the arrival of the AMS team the DIR-MED decided to reduce the administration of antidotes to 1 auto-injector per severely intoxicated victim. In accordance with Rodriguez and McClellan’s PKPD model, the probability of a decrease in IP of this act was only 70% instead of 85% in victims with a lethal sarin exposure (23). For the final step, we tried to identify the factors contributing to mortality. We analyzed the locations within the medical response chain where victims died. Each victim’s location of death was determined by the last step in the medical response process they had entered before death. These locations include the subway station (waiting for evacuation), triage queue (waiting for triage), decontamination queue (i.e., after being triaged), FMP transport (after decontamination, only in Stay&Play), and hospital transport. Hospital transport deaths are defined as victims dying while waiting for transport to the hospital (i.e., after FMP treatment in Stay&Play or after completing decontamination in Scoop&Run).

**Table 2:** Summarized overview of the application of the STRESS checklist to the model.

Category	Checklist item	Present simulation model
Objectives	Purpose of the model	Assess the impact of antidote availability, AMS team timing, and evacuation policy on mortality.
	Model outputs	Number of preventable deaths among 25 salvageable victims, stratified by step and location.
Experimentation	Experimentation aims	Four studies varying AMS team arrival, antidotes, evacuation, and resource bottlenecks.
Logic	Base model overview	Sequential victim flow from exposure through triage, treatment, and hospital transport.
	Base model logic	DES structure with discrete evacuation, triage, decontamination, AMS team, (optional FMP) and transport processes.
	Scenario logic	Each experiment modifies arrival times, antidote counts, policy, or resource configurations.
	Algorithms	Priority queuing, triage survival logic, transport assignment, and dynamic resource control.
	Components	Victims, medical staff, hospitals, ambulances, and facilities with capacity and routing rules.
Data	Data sources	Military and civilian trauma models, expert input, OpenStreetMap, and pharmacokinetic data.
	Pre-processing	Synthetic generation of victim profiles with standardized formats; no imputation needed.
	Input parameters	Fixed and stochasticly varied inputs for arrival times, triage durations, antidote levels, and transport delays.
	Assumptions	Ideal task execution, no expectant category, fixed treatment effects, and prehospital mortality only.
Experimentation	Experimentation aims	Four studies varying AMS team arrival, antidotes, evacuation, and resource bottlenecks.
	Initialization	Initialization at the start of exposure with parameters in table 3. Terminating system; simulation ends after last victim reaches home, outpatient clinic, hospital or dies.
	Run length	Dynamic run length based on completion of all prehospital processes; measured in minutes.
	Estimation approach	Five replications per scenario; confidence intervals and variance comparison used.
Implementation	Software / programming language	Julia 1.8 with SimJulia 0.8.2; mapping with OpenStreetMap
	Random sampling	Mersenne Twister with a fixed seed per replication number.
	Model execution	Process-interaction with priority-based queues and survival checks during resource allocation.
	System specification	2020 iMac with a 3,1 GHz 6-Core Intel Core i5 and 24GB DDR4 Ram
Code access	Computer model sharing	Modeling information available upon request from corresponding author

The full checklist is available in the Supplementary materials (35).

**Table 3:** Experimental design parameters.

Parameter		Values
Simulator replications	All analyses	5 replications per parameter combination
AMS team arrival times (in minutes)	Arrival time analysis	0, 5–25 (2-min intervals)
	Quantitative Antidote analysis	9, 11, 13 and 15 min
	Bottleneck analysis	9, 11, 13 and 15 min
	Auto-injector decrease analysis	0, 5–25 (2-min intervals)
Antidote doses (auto-injectors)	Arrival time analysis	10 (15), 20 (30), 40 (60)
	Quantitative Antidote analysis	0(0), 10(15), 20(30), 40(60), 60(90), 415(517)
	Bottleneck analysis	10 (15), 20 (30), 40 (60)
	Auto-injector decrease analysis	10 (15), 20 (30), 40 (60)
Evacuation policies	All analyses	Stay&Play and Scoop&Run
Victim intoxication levels	All scenarios	Severe: 34 non-lethal and 17 lethal. Non-severe: 364 Moderate and 411 mild Unaffected: 168
Salvageable victims	All scenarios	16 chemical, 8 traumatic and 1 mixed chemical/trauma

## 3 Results

**Figure 2:**
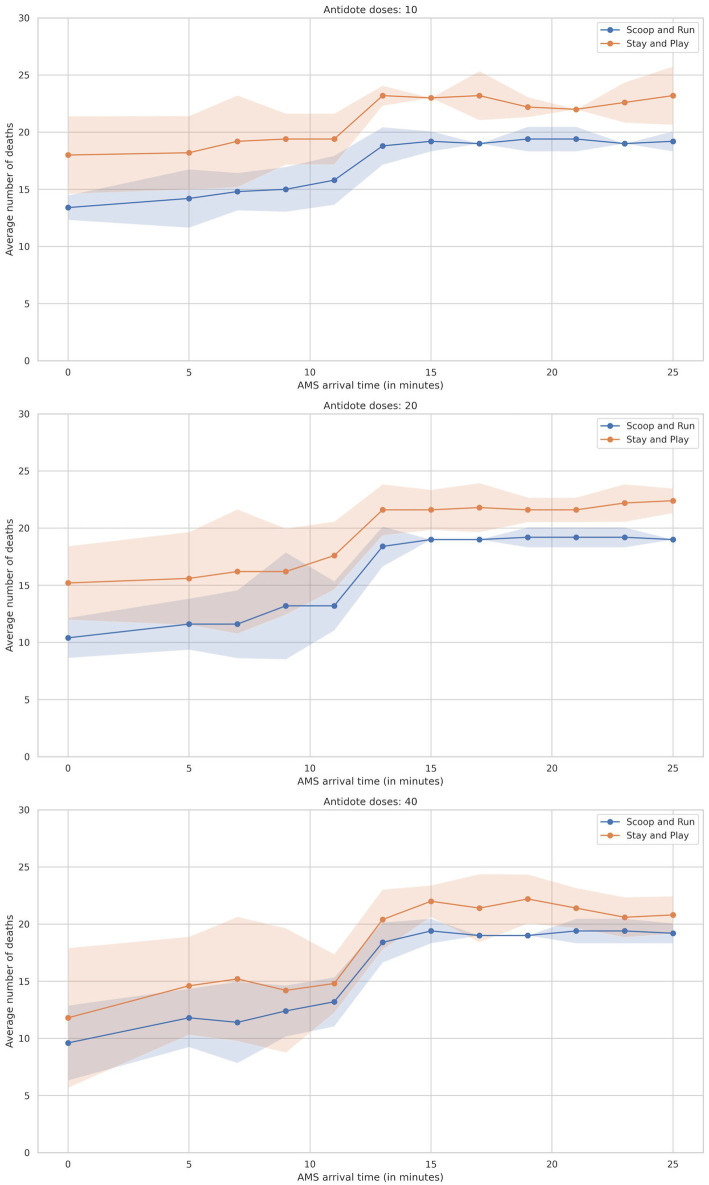
Plot of average number of preventable deaths by AMS team arrival time divided by evacuation policy, split by the number of antidotes. Shaded areas represent the 95% confidence interval.

**Figure 3:**
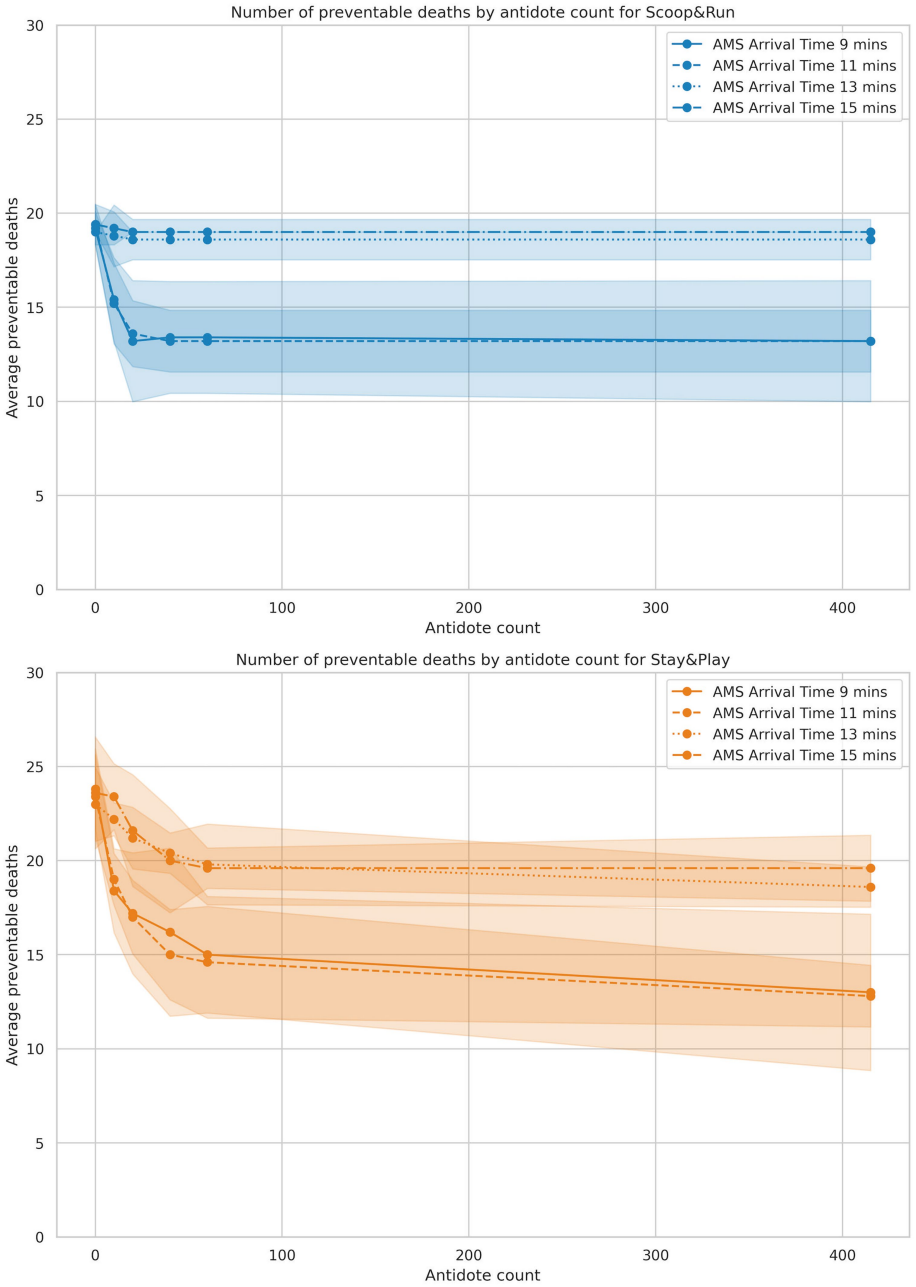
Results of the antidotes analysis, split by policy. Antidote doses are on the X-axis, and average number of preventable deaths is represented on the Y-axis. Shaded areas represent the 95% confidence intervals.

**Figure 4:**
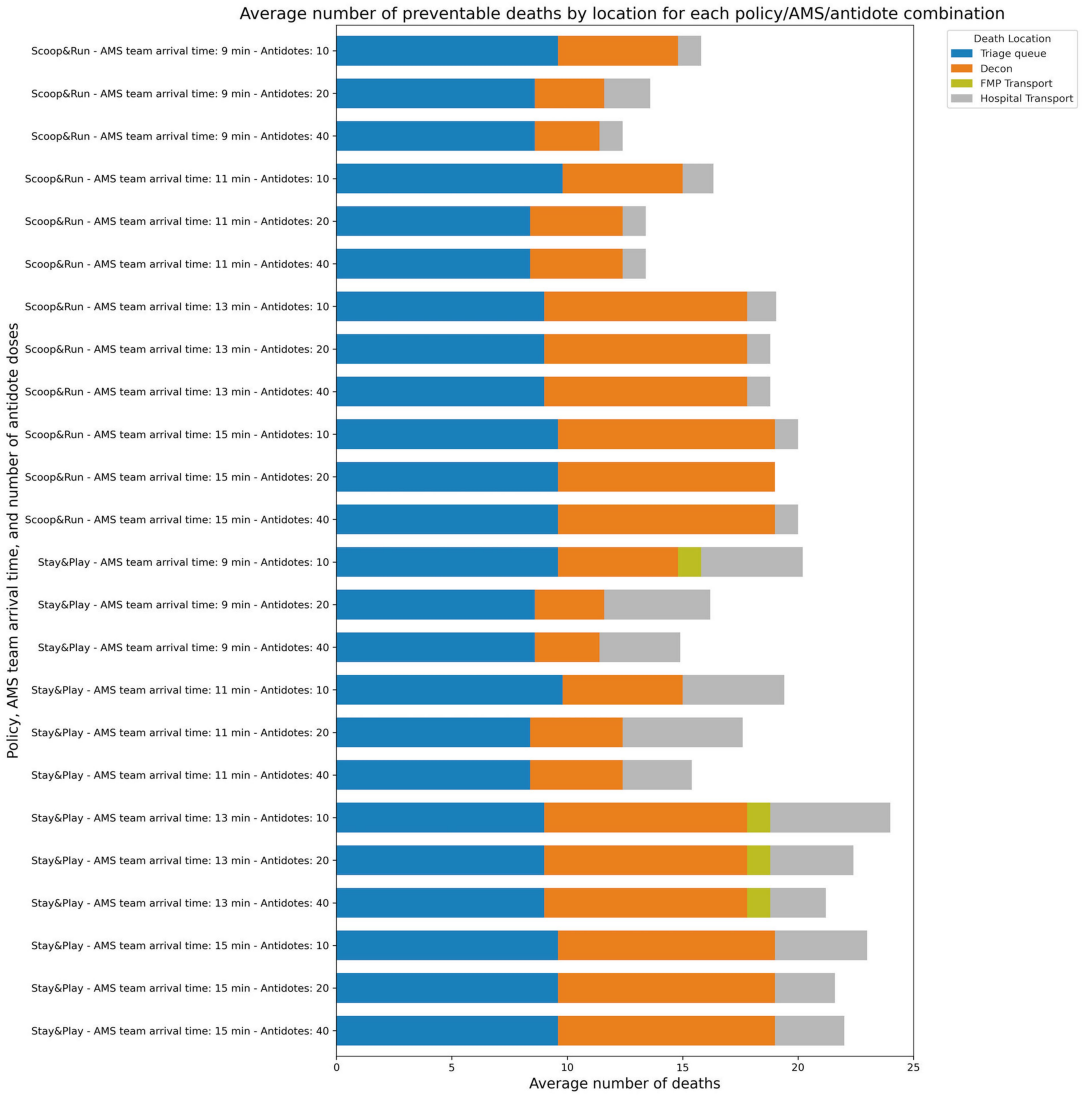
Average number of preventable deaths by evacuation policy, location and number of antidote doses available.

Across all scenarios, the number of preventable deaths among salvageable victims—defined as those who could survive with timely and adequate treatment—was clearly influenced by AMS team arrival time, antidote availability, and evacuation policy. Each scenario included 25 salvageable victims, allowing death outcomes to be reported as both an average number of preventable deaths and a percentage of this fixed group of salvageble victims. In the text below, the percentages between brackets reflect the proportion of salvageable victims. Overall, the number of preventable deaths ranged from 8.0 (32%) in the most favorable scenario (Scoop&Run, 40 antidote doses, 5-min AMS team arrival) to 23.8 (95.2%) in the least favorable case (Stay&Play, no antidotes, 11-min arrival). Key results are summarized below and supported by Figures 2–4. Tables of full data with standard deviations are available in the Supplementary material.

### 3.1 Effect of AMS team arrival time

The number of preventable deaths among salvageable victims increased substantially with delayed AMS team arrival across both evacuation policies. For instance, preventable deaths ranged from 11.4 (45.6%) under the Scoop&Run policy with a 7-min AMS team arrival and 40 antidote doses, to 23.2 (92.8%) under the Stay&Play policy with a 25-min arrival and 10 antidote doses.

Figure 2 illustrates this relationship, plotting death outcomes against AMS team arrival time and stratified by antidote availability. The mortality curve followed a sigmoid pattern: when arrival occurred within 9 min, preventable deaths remained low and stable. However, as arrival times reached 10–15 min, preventable deaths rose sharply—especially in simulations with only 10 or 20 antidote doses. Beyond 21 min, the rate of increase declined, indicating a plateau in the mortality curve.

The Scoop&Run policy consistently resulted in fewer deaths than Stay&Play, particularly when AMS team arrival occurred before 11 min. At short arrival times, Stay&Play exhibited elevated baseline preventable deaths and maintained this disadvantage across most time points.

While increasing the number of available antidotes did not uniformly reduce deaths across all scenarios, higher availability tended to reduce baseline preventable death levels and mitigate the sharp rise seen at moderate delays. For example, with 40 antidote doses, AMS team arrival within the first 5 min led to substantially fewer deaths compared to simulations with only 10 doses. However, even with ample antidote stocks, the steep rise in deaths at 10–15 min persisted—highlighting the critical role of rapid AMS team deployment.

### 3.2 Effect of antidote dose quantity and auto-injector reduction

Antidote availability had a substantial effect on the number of preventable deaths among salvageable victims, particularly under conditions of moderate AMS team arrival delay. Across all scenarios, average preventable deaths ranged from 12.8 (51.2%) in the most favorable antidote condition (Stay&Play, 9-min AMS team arrival, 100 antidote doses) to 23.8 (95.2%) in the most constrained scenario (Stay&Play, 11-min arrival, no antidotes). The results of the antidote auto-injector reduction analysis failed to show a significant difference when compared to the first experiment baseline (see Supplementary material for the exact data and statistical analysis). Further analysis showed that at the time this decision was taken, most salvageable patients had either already been decontaminated or had died due to lack of treatment.

Figure 3 presents death outcomes as a function of available antidote doses, stratified by AMS team arrival time and evacuation policy. In the Scoop&Run policy, preventable deaths decreased sharply as the number of antidote doses increased, but the benefit of additional doses diminished beyond approximately 40–60 doses, with curves flattening toward a plateau. Earlier AMS team arrival (11 min or less) consistently corresponded to fewer preventable deaths than later arrival times (greater than 13 min), regardless of antidote levels.

Under the Stay&Play policy, baseline preventable deaths were higher at low antidote levels compared to Scoop&Run. However, preventable deaths also decreased rapidly as antidote availability increased. The steep slope in this low-dose range indicates that outcomes under Stay&Play were especially sensitive to antidote availability. At higher antidote doses (60 or more), death curves across all AMS team arrival times converged to similar levels, suggesting a ceiling effect in treatment efficacy when sufficient antidotes are available.

Overall, Scoop&Run resulted in fewer preventable deaths than Stay&Play across all antidote doses. However, this difference diminished at the highest antidote levels, where both policies produced comparable outcomes. These findings underscore the interaction between antidote availability and response time: while timely AMS team arrival is consistently beneficial, substantial antidote availability can partially compensate for delayed arrival—especially under the Stay&Play policy.

### 3.3 Place of death in the prehospital response chain

Figure 4 shows the distribution of averaged death counts across different stages of the prehospital response chain, stratified by AMS team arrival time, antidote doses, and evacuation policy. Preventable deaths were most frequently concentrated in the FMP and transport waiting areas, with significantly more preventable deaths occurring in the Stay&Play policy compared to Scoop&Run.

Across all scenarios, the principal locations of preventable deaths were the triage queue in the CCP, decontamination area, and during delays in FMP or hospital transport. No preventable deaths were observed at the subway station. Preventable deaths at the triage queue remained relatively constant across all scenarios, unaffected by either antidote levels or AMS team arrival time. In contrast, preventable deaths during decontamination decreased in a dose-dependent manner with increasing antidote availability. However, this effect was markedly diminished when AMS team arrival was delayed beyond 11 min, indicating that timely delivery of stabilizing care is essential to realizing the benefits of antidote availability.

The largest difference in preventable deaths between evacuation policies was attributed to deaths occurring at the FMP while waiting for hospital transport. This suggests a systemic bottleneck specific to the Stay&Play configuration. To evaluate this hypothesis, simulations were re-run with enhanced transport capacity: doubling the number of MMTs, doubling FMP minimum staffing, quadrupling ambulance capacity, and assigning twice as many ambulances for CCP-to-FMP transfers under the Stay&Play policy. The outcomes of this analysis are shown in Supplementary Figure 1A.

In these enhanced-capacity scenarios, preventable deaths decreased significantly. The lowest average number of preventable deaths was 8.0 (32%) under the Scoop&Run policy (40 antidote doses, 5-min AMS team arrival), and the highest was 20.6 (82.4%) under Stay&Play (20 antidotes, 23-min arrival). Most importantly, preventable deaths associated with transport delays to the FMP or hospital were markedly reduced. For the Stay&Play policy, this led to a substantial, antidote-dependent decrease in total preventable deaths, particularly when AMS team arrival occurred within 10 min—effectively eliminating the difference in preventable death outcomes between Stay&Play and Scoop&Run. A similar but less pronounced trend was observed in the Scoop&Run policy, especially at higher antidote levels.

The exact average number of preventable deaths with standard deviation for combinations of every AMS team arrival time, antidote dose and evacuation policy are available in the Supplementary material to this publication.

## 4 Discussion

The results highlight the importance of minimizing total prehospital time and ensuring the rapid antidote availability in managing urban chemical MCIs. Three main operational factors were shown to determine mortality among salvageable victims: AMS team arrival time, antidote availability, and evacuation policy.

AMS team arrival time emerged as the strongest predictor of preventable mortality. Simulations revealed a sigmoid-shaped mortality curve: the number of preventable deaths remained low when AMS teams arrived within 11 min but rose sharply between 11 and 15 min. This reflects the expected progression of symptoms after sarin exposure, where untreated victims typically die within 20 to 25 min. Under higher exposure concentrations or increased minute volume, this window shortens even further (36). Considering a typical 10-min deployment delay, this leaves only a brief and critical window for LSI. It has been demonstrated that total prehospital times often exceeds several hours in trauma-related MCIs (37). AMS teams serve as a time-sensitive bridge to survival, allowing victims to endure the rest of the prolonged prehospital sequence, including decontamination, optional FMP stabilization, and hospital transport.

Evacuation strategy also influenced outcomes. Scoop&Run consistently resulted in fewer preventable deaths than the Stay&Play approach, where more extensive treatment is provided before transport. The advantage of Scoop&Run was most pronounced when AMS teams arrived quickly, suggesting that reducing scene time without compromising basic stabilization may offer the best balance. In scenarios where transport capacity was enhanced, preventable deaths further decreased highlighting that operational improvements can significantly influence outcomes. The interaction between antidotes, rapid AMS arrival, and transport logistics suggests a synergistic effect, where early interventions set the stage for improved survival throughout the rest of the care continuum.

Antidote availability exerted a dose-dependent effect on the number of preventable deaths, particularly in scenarios with moderate AMS team arrival delays. While increasing antidote doses from 0 to 40 significantly reduced preventable deaths, benefits diminished beyond 60 doses, reflecting a ceiling effect in treatment efficacy in this scenario. While antidote availability remains essential, the model shows that the benefit of antidotes is significantly diminished when they arrive later than 11 min.

Analysis of the location of deaths revealed that delays in transport to the FMP or to the hospital are the main bottlenecks. No preventable deaths occurred at the initial exposure site, suggesting that delays in stabilizing care were the primary cause of preventable deaths. Between 40 and 50% of deaths among salvageable victims were attributable to modifiable operational factors such as activation delay, resource arrival time, setup duration, and decontamination speed.

Several findings from our simulation experiments reflect key patterns reported in major nerve agent incidents, including the 1995 Tokyo sarin attack, the 2013 Ghouta attack, and the 2017 Khan Shaykhun attack. In Tokyo, Okumura et al. observed that a lack of prehospital antidotes, limited recognition of nerve agent symptoms, and absent EMS protocols contributed to avoidable morbidity and mortality. Most victims self-presented to hospitals, overwhelming facilities and delaying care (38, 39). Similar challenges were observed in Syria. In Khan Shaykhun early administration of basic supportive care (namely oxygen and atropine) was sufficient to ensure survival in all admitted patients, despite minimal staffing, no pralidoxime, and lack of advanced diagnostics (40). In Ghouta, victims were treated without protective equipment or monitoring and relied on in-hospital treatment with atropine due to the absence of prehospital auto-injectors and oximes. Critically injured patients likely died from inadequate airway support and delayed treatment (41). Across all three cases, the absence of prehospital CBRN preparedness and the inability to deliver antidotes rapidly and consistently were the main contributors to preventable deaths. These reports suggest that early, adequate antidote administration and streamlined evacuation are essential for reducing mortality even when advanced interventions are unavailable. However effective antidote use during MCIs also depends on the ability of responders to correctly dose and administer treatment under stressful conditions (42). Our simulation supports the conclusions drawn from these reports by showing that the timely arrival of a well-trained and well-equipped prehospital CBRN team combined with rapid transport to nearby hospitals, will likely reduce morbidity and mortality in nerve agent incidents.

Our findings also raise questions about the appropriateness of storing nerve agent antidotes in centralized stockpiles. Given the narrow therapeutic window, centrally stored antidotes may not be accessible in time to influence outcomes during the critical early phase of a chemical MCI. Decentralizing antidote distribution and placing them with first responders such as EMS and HAZMAT teams may significantly reduce time to administration and improve survival (43). Our results reinforce this recommendation by demonstrating that the benefit of antidotes is strongly time-dependent and largely lost when administration is delayed.

### 4.1 Limitations

This simulation assumes perfect execution of tasks by all responders and does not account for the chaos and unpredictability inherent in MCIs. Although some stochastic variation in timing is included, treatment outcomes are deterministic except for the probability of antidote application. The model does not account for provider skill variation, resource fatigue, or miscommunication. Although they may significantly impact scene dynamics, behavioral variables such as mass psychogenic illness, ‘worried well,’ or victims bypassing EMS transport are also excluded on the grounds of insufficient data availability for reliable modeling (38, 39, 44–46).

Currently, the simulator assumes a uniform treatment timing and outcome across all victims. This simplification was necessary due to the lack of validated evidence models capable of predicting how individual interventions affect a victim’s health trajectory. Although data on treatment durations exist in the literature (and correlate well to the data used in this simulation), they cannot yet support reliable modeling of patient-specific effects or resource allocations (47). Incorporating treatment effects and resources in future models would improve realism, though the impact on overall scene time is likely to be small compared to the broader delays typical to MCI care (37).

Furthermore, the model does not incorporate in-hospital treatment, ICU demand, ventilator capacity, or long-term sequelae of nerve agent exposure due to data limitations. Many assumptions are based on expert opinion due to a lack of available data. Consequently, prehospital deaths within the simulated environment are a useful but partial outcome measure. If more data would be available allowing the connection between the prehospital and in-hospital treatments and outcome, long-term morbidity and mortality (e.g., organophosphate induced delayed neuropathy) would be a better outcome parameter.

It should also be noted that the generalizability of these findings is limited by the specificity of the modeled scenario. The simulation is based on the structure, capacities, legal framework and operational protocols of the Belgian EMS, particularly as applied to the Brussels urban context. As such, conclusions drawn from this study may not be directly transferable to regions with substantially different EMS organization, antidote application and distribution strategies, or hospital network configurations.

### 4.2 Future research directions

Due to the organization of the Belgium prehospital system, the arrival of antidotes and the arrival of the AMS team are tied together, which does not allow us to independently examine the effects of antidote application outside of the AMS care. Future work should explore the feasibility and impact of expanding antidote administration capabilities to frontline responders, for example HAZMAT teams. We hypothesize that antidote delivery by the HAZMAT team could extend the window of salvageability and even further decrease the number of preventable deaths. On the other end of the response chain, in hospital modeling is also essential to capture the full continuum of care, particularly ICU burden and long-term neurological outcomes as mentioned in the limitations above.

Another priority is the development of a prehospital treatment model for MCIs that incorporates treatment-specific resources, individualized intervention times, and injury-specific treatment effects on victim health states. Dynamically assigning intervention times based on individual victim profiles could allow for more granular modeling of medical workflows and resource demands, helping to overcome the tendency of underestimation of MCI prehospital treatment times (47).

Finally, integrating behavioral dynamics into future simulations such as spontaneous evacuation, bystander intervention, or EMS overload from non-exposed individuals can also improve realism and planning relevance.

## 5 Conclusion

This simulation study, based on a sarin release scenario in a subway station demonstrates that survival among salvageable victims is highly dependent on operational timing specifically, the prompt arrival of antidote-equipped AMS teams, timely antidote administration, and minimization of prehospital delays. The results confirm that a specialized AMS team and coordinated prehospital response can significantly reduce mortality in nerve agent MCIs, especially when combined with a Scoop&Run approach that minimizes on-scene time. While the findings are scenario-specific, they highlight actionable priorities for chemical incident preparedness: reducing AMS team deployment delays, ensuring timely antidote delivery, and selecting evacuation strategies that avoid bottlenecks. Together, these operational priorities provide a practical foundation for strengthening urban chemical MCI preparedness and response.

## Data Availability

The raw data supporting the conclusions of this article will be made available by the authors, without undue reservation.
