# Supplementary material for: Impact of antidote quantity, timing and prehospital strategies in nerve agent mass casualty events: a simulation study
Source: Front Public Health. 2025 Aug 26;13:1640554. doi: 10.3389/fpubh.2025.1640554 (PMC12417396; doi:10.3389/fpubh.2025.1640554)
Supplement: Supplementary file 1 [file Data_Sheet_1.docx]

Supplementary Material

# Introduction

In this appendix we report additional tables and figures that we feel did not meet the criteria for inclusion in the main article, but will aid in interpreting the results and supporting our conclusion.

# Three to 1 auto-injector reduction for severe intoxications

During development of the scenario, a reduction in the treatment of severely intoxicated victims from 3 to 1 auto-injector application was suggested by one of the subject matter experts. This was modelled as an operational decision taken by the DIR-MED within 10 minutes of both the DIR-MED and the AMS team arriving on-site, to incorporate the decision and communication delays. The application of one antidote auto-injector only has 70% effectiveness in reversing the lethality, as opposed to 85% for 3 auto-injectors in lethally intoxicated victims based on the PKPD model by McClellan and Rodriguez**.** ^19^ The rationale behind this decision is to increase the number of victims that receive the antidotes, assuming that incomplete treatment is better than no treatment. We ran this in the simulation but found no significant difference between the 3 auto-injectors and 1 auto-injector application (see Supplementary table A2 and supplementary figure A3 and A4). We modelled this decision, but results suggest the decision will be taken too late. Due to the combination of arrival times, delays in the decision-making process and auto-injector handover, this decision goes into effect when most of the victims who would potentially benefit from the freed auto-injectors are already deceased or have already progressed past the decontamination station either to the FMP or to hospital transport.

# Supplementary Figures

**Supplementary Figure A**1 is a plot of average number of preventable deaths by AMS team arrival time divided by evacuation policy, split by the number of antidotes. Shaded areas represent the 95% confidence interval. The lighter-coloured lines and shades represent the simulation replications where the bottlenecks were counteracted. This was achieved by doubling the number of MMTs and quadrupling the number of ambulances, as well as doubling the FMP minimum staffing and number of ambulances for transport between decontamination and the FMP.

**Supplementary Figure A2** is the equivalent of figure 4 in the main publication but for the simulation experiment where the number of ambulances and MMTs are artificially increased. It shows the distribution of preventable deaths by location for several combinations of AMS team arrival time.

**Supplementary Figure A3** displays of average number of preventable deaths by AMS team arrival time divided by evacuation policy, split by the number of antidotes. Shaded areas represent the 95% confidence interval. The lighter coloured lines represent the intervention group where the DIR-MED orders a rationing of the antidote auto-injectors applied at SALT triage or by the AMS team from 3 to 1 auto-injector per victim.

**Supplementary Figure A4** is a boxplot of mean last auto-injector administration times, grouped by AMS team arrival time and number of auto-injector. It demonstrates that this decision is taking when most auto-injectors are spent, except in the simulation replications with the highest number of antidotes.


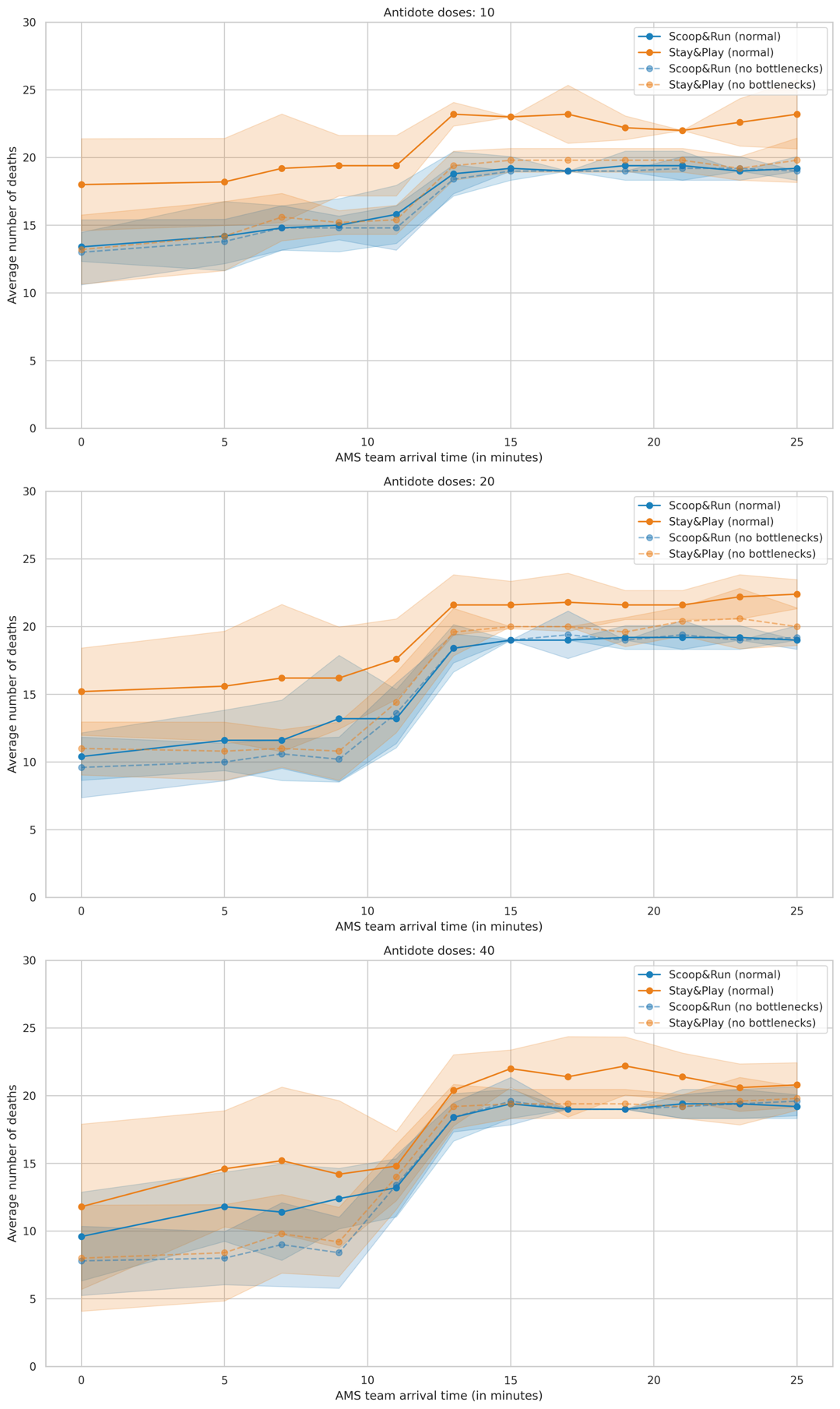


**Supplementary Figure A**1: Plot of average number of preventable deaths by AMS team arrival time divided by evacuation policy, split by the number of antidote doses. Shaded areas represent the 95% confidence interval. The lighter-coloured dashed lines represent the simulation replications where the bottlenecks were counteracted.


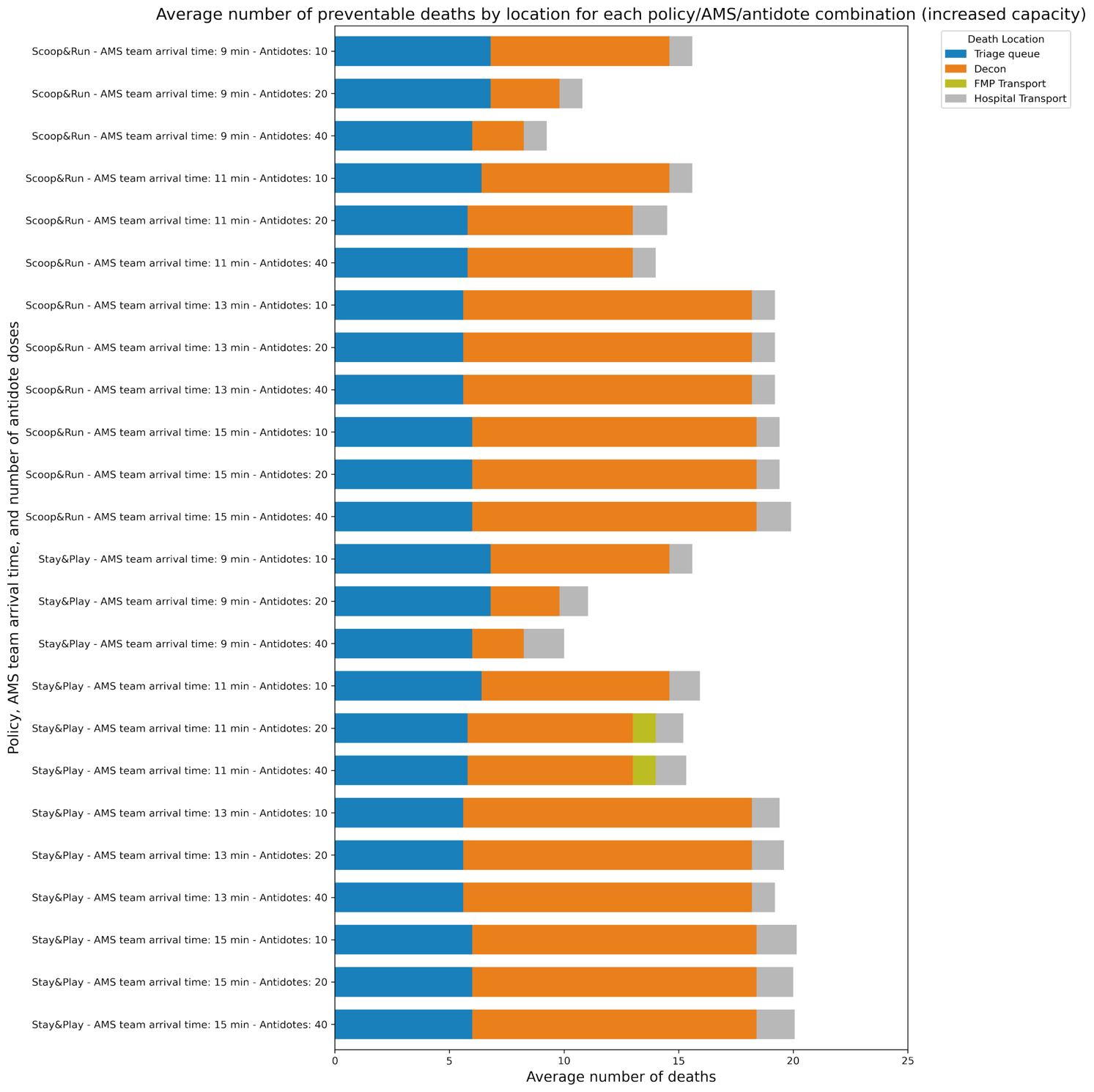
 **Figure Supplementary Figure A2:** Average number of preventable deaths by evacuation policy, location and number of antidote doses available for the simulation runs with doubled FMP transport capacity (if applicable), doubled MMT capacity and quadrupled number of ambulances.


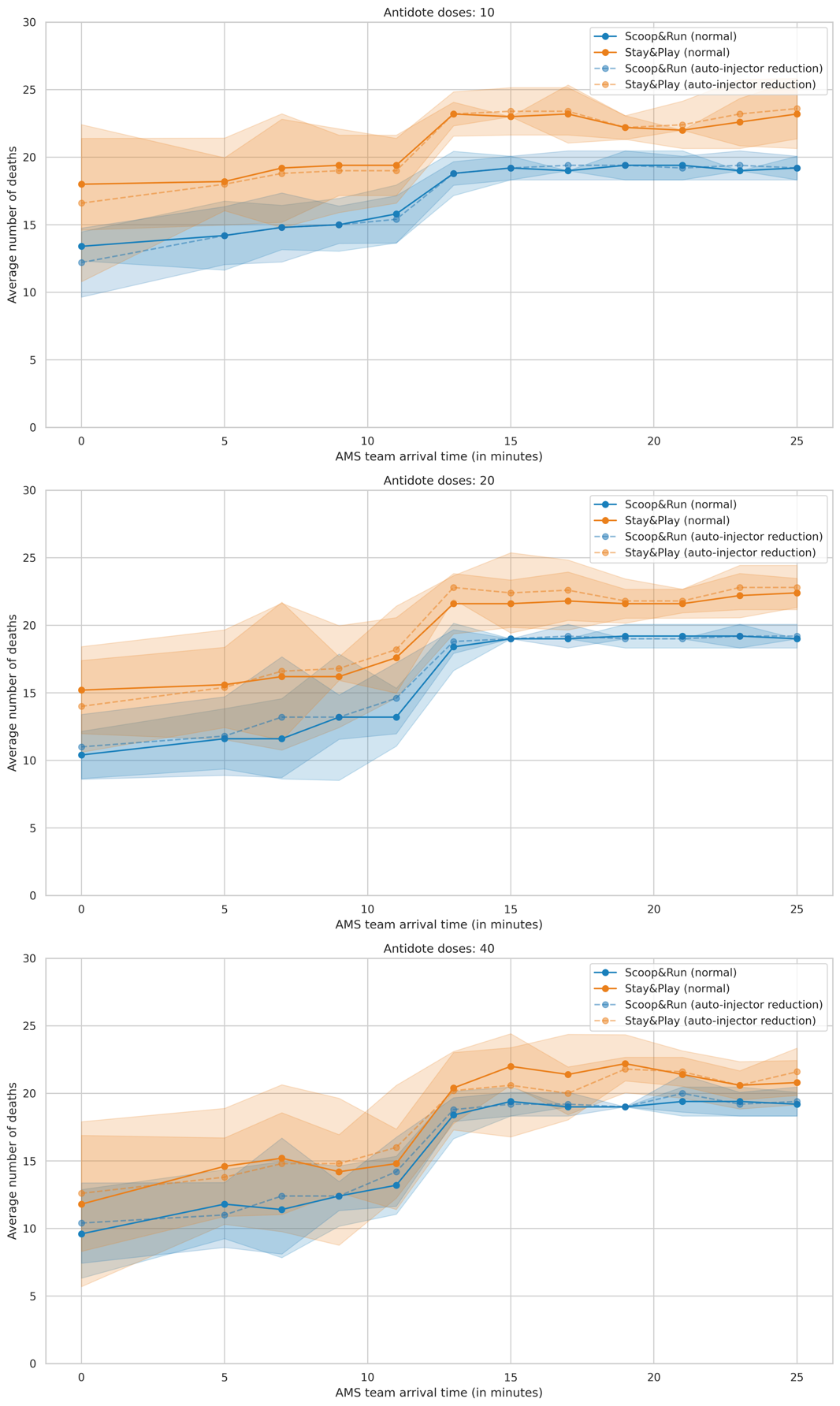


**Supplementary Figure A3:** Plot of average number of preventable deaths by AMS team arrival time divided by evacuation policy, split by the number of antidotes. Shaded areas represent the 95% confidence interval. The lighter-coloured dashed lines represent the intervention group where the DIR-MED orders a rationing of the antidote auto-injectors applied at SALT triage or by the AMS team from 3 to 1 auto-injector per victim.


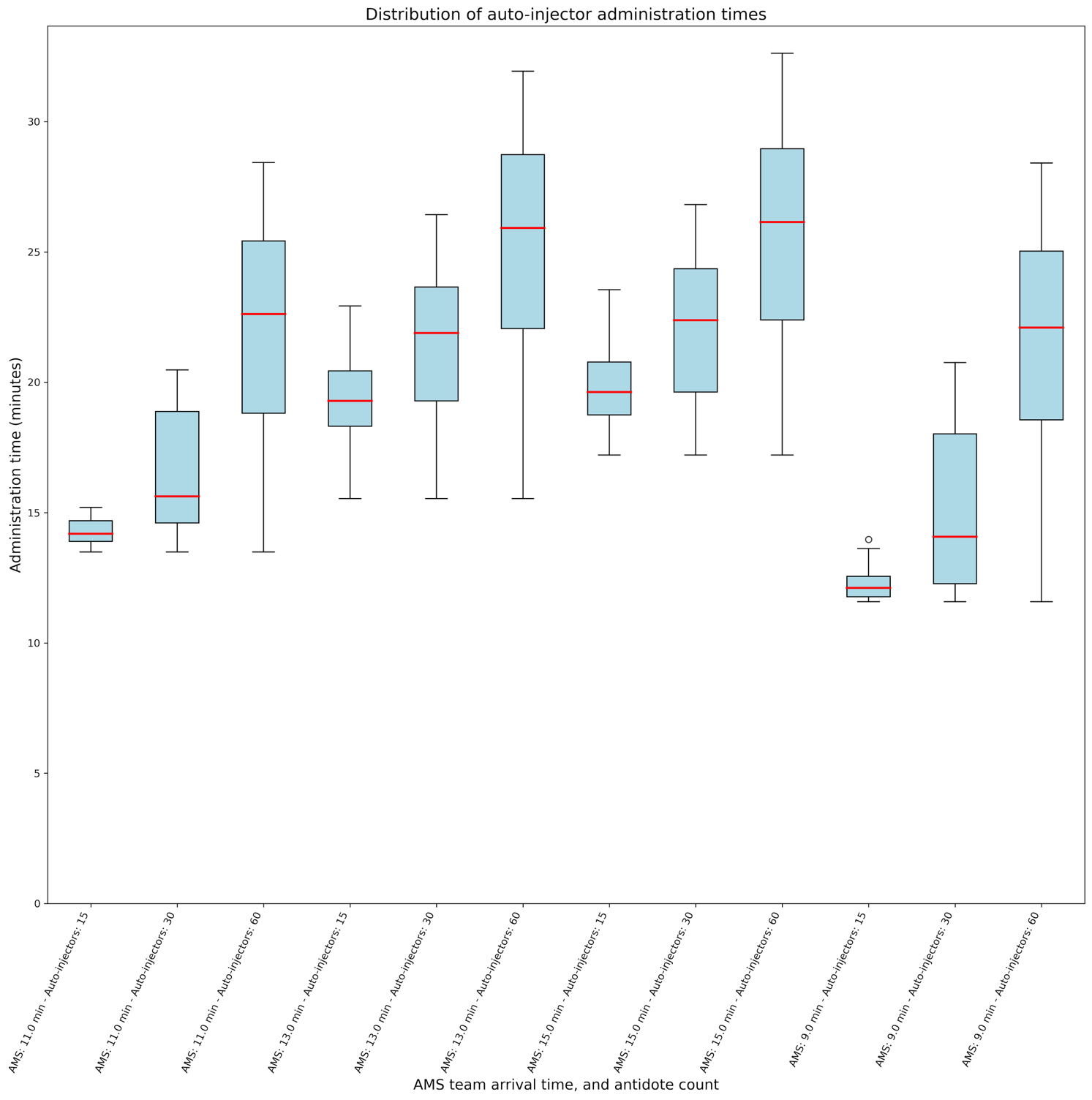


**Supplementary Figure A4:** Boxplot of auto-injector administration times by AMS team arrival time and number of auto-injectors.

**4 Supplementary Tables**

**Supplementary Table A1** displays the average number of preventable deaths by AMS team arrival time, antidote doses and evacuation policy. SD represents the standard deviation, min the lowest number of preventable deaths and max the highest number of preventable deaths for that parameter combination. This data is graphically represented in figure 2 of the article.

**Supplementary Table A2** shows the results of a linear regression analysis to compare the auto-injector reduction intervention to the control group without this intervention. This model explains about 77% of the variability in total preventable deaths (R-squared = 0.764) and is highly statistically significant overall (F-statistic p-value < 0.000). The significant predictors are evacuation policy, AMS team arrival time, number of antidote doses, and the two-way interaction between AMS team arrival time and antidote doses. The Stay&Play policy and AMS team arrival time both increase the number of preventable deaths, whereas antidote doses has a decreasing effect on the number of preventable deaths. The auto-injector rationing variable and its interactions are not statistically significant. This shows that this intervention has no significant effect on the number of preventable deaths.

**Supplementary Table A3** displays the mean auto-injector administration times for the standard group (i.e. no reduction in auto-injectors given). It shows the mean time of administration of the last auto-injector, as well as the standard deviation (SD), fastest and slowest time of administration of the last auto-injector. This data is also represented in a boxplot in figure A4. Both policies have identical values as they only affect the results downstream from the intervention. When combining this data with the hypothetical decision to reduce the number of auto-injectors given to unconscious victims from 3 to 1 - which is taken 10 minutes after AMS team arrival time - it is clear this decision can only have a possible effect in the cases where the number of antidotes is higher than 60. This is because the mean time of final auto-injector application lies before the decision time in cases with 30 or fewer auto-injectors. It also should be noted that victim level of consciousness is based on the health state parameter, which is affected by both the severity of the intoxication as well as the effects of their traumatic injuries. This is combination is unique and specific to each victim, and the timing of this phenomenon varies due to stochastic variation in treatment and transport times. Most severely injured victims will also have progressed through the response chain by the time the antidote reduction takes effect. All of this results in large portion of the antidotes being given to the moderately severe and severe (but not lethal) intoxications and not significantly affecting our primary endpoint.

**Supplementary Table A1**: Average number of preventable deaths by AMS team arrival time, antidote doses and evacuation policy. SD represents the standard deviation, min the lowest number of preventable deaths in this replication series, and max the highest number of preventable deaths.

| Arrival Time | Antidote doses | Evacuation policy | mean | SD | min | max |
| --- | --- | --- | --- | --- | --- | --- |
| **5 minutes** | **10** | **Scoop&Run** | 14.2 | 1.30 | 13 | 16 |
|  |  | **Stay&Play** | 18.2 | 1.64 | 16 | 20 |
|  | **20** | **Scoop&Run** | 11.6 | 1.14 | 10 | 13 |
|  |  | **Stay&Play** | 15.6 | 2.07 | 14 | 19 |
|  | **40** | **Scoop&Run** | 11.8 | 1.30 | 10 | 13 |
|  |  | **Stay&Play** | 14.6 | 2.19 | 11 | 17 |
| **7 minutes** | **10** | **Scoop&Run** | 14.8 | 0.84 | 14 | 16 |
|  |  | **Stay&Play** | 19.2 | 2.05 | 17 | 21 |
|  | **20** | **Scoop&Run** | 11.6 | 1.52 | 10 | 14 |
|  |  | **Stay&Play** | 16.2 | 2.77 | 13 | 20 |
|  | **40** | **Scoop&Run** | 11.4 | 1.82 | 9 | 14 |
|  |  | **Stay&Play** | 15.2 | 2.77 | 13 | 20 |
| **9 minutes** | **10** | **Scoop&Run** | 15.0 | 1.00 | 14 | 16 |
|  |  | **Stay&Play** | 19.4 | 1.14 | 18 | 21 |
|  | **20** | **Scoop&Run** | 13.2 | 2.39 | 11 | 17 |
|  |  | **Stay&Play** | 16.2 | 1.92 | 14 | 19 |
|  | **40** | **Scoop&Run** | 12.4 | 1.14 | 11 | 14 |
|  |  | **Stay&Play** | 14.2 | 2.77 | 11 | 17 |
| **11 minutes** | **10** | **Scoop&Run** | 15.8 | 1.10 | 14 | 17 |
|  |  | **Stay&Play** | 19.4 | 1.14 | 18 | 21 |
|  | **20** | **Scoop&Run** | 13.2 | 1.10 | 12 | 14 |
|  |  | **Stay&Play** | 17.6 | 1.52 | 16 | 19 |
|  | **40** | **Scoop&Run** | 13.2 | 1.10 | 12 | 14 |
|  |  | **Stay&Play** | 14.8 | 1.30 | 13 | 16 |
| **13 minutes** | **10** | **Scoop&Run** | 18.8 | 0.84 | 18 | 20 |
|  |  | **Stay&Play** | 23.2 | 0.45 | 23 | 24 |
|  | **20** | **Scoop&Run** | 18.4 | 0.89 | 17 | 19 |
|  |  | **Stay&Play** | 21.6 | 1.14 | 20 | 23 |
|  | **40** | **Scoop&Run** | 18.4 | 0.89 | 17 | 19 |
|  |  | **Stay&Play** | 20.4 | 1.34 | 18 | 21 |

| Arrival Time | Antidote doses | Policy | mean | SD | min | max |
| --- | --- | --- | --- | --- | --- | --- |
| **15 minutes** | **10** | **Scoop&Run** | 19.2 | 0.45 | 19 | 20 |
|  |  | **Stay&Play** | 23.0 | 0.00 | 23 | 23 |
|  | **20** | **Scoop&Run** | 19.0 | 0.00 | 19 | 19 |
|  |  | **Stay&Play** | 21.6 | 0.89 | 21 | 23 |
|  | **40** | **Scoop&Run** | 19.4 | 0.55 | 19 | 20 |
|  |  | **Stay&Play** | 22.0 | 0.71 | 21 | 23 |
| **17 minutes** | **10** | **Scoop&Run** | 19.0 | 0.00 | 19 | 19 |
|  |  | **Stay&Play** | 23.2 | 1.10 | 22 | 25 |
|  | **20** | **Scoop&Run** | 19.0 | 0.00 | 19 | 19 |
|  |  | **Stay&Play** | 21.8 | 1.10 | 20 | 23 |
|  | **40** | **Scoop&Run** | 19.0 | 0.00 | 19 | 19 |
|  |  | **Stay&Play** | 21.4 | 1.52 | 20 | 24 |
| **19 minutes** | **10** | **Scoop&Run** | 19.4 | 0.55 | 19 | 20 |
|  |  | **Stay&Play** | 22.2 | 0.45 | 22 | 23 |
|  | **20** | **Scoop&Run** | 19.2 | 0.45 | 19 | 20 |
|  |  | **Stay&Play** | 21.6 | 0.55 | 21 | 22 |
|  | **40** | **Scoop&Run** | 19.0 | 0.00 | 19 | 19 |
|  |  | **Stay&Play** | 22.2 | 1.10 | 21 | 24 |
| **21 minutes** | **10** | **Scoop&Run** | 19.4 | 0.55 | 19 | 20 |
|  |  | **Stay&Play** | 22.0 | 0.00 | 22 | 22 |
|  | **20** | **Scoop&Run** | 19.2 | 0.45 | 19 | 20 |
|  |  | **Stay&Play** | 21.6 | 0.55 | 21 | 22 |
|  | **40** | **Scoop&Run** | 19.4 | 0.55 | 19 | 20 |
|  |  | **Stay&Play** | 21.4 | 0.89 | 20 | 22 |
| **23 minutes** | **10** | **Scoop&Run** | 19.0 | 0.00 | 19 | 19 |
|  |  | **Stay&Play** | 22.6 | 0.89 | 22 | 24 |
|  | **20** | **Scoop&Run** | 19.2 | 0.45 | 19 | 20 |
|  |  | **Stay&Play** | 22.2 | 0.84 | 21 | 23 |
|  | **40** | **Scoop&Run** | 19.4 | 0.55 | 19 | 20 |
|  |  | **Stay&Play** | 20.6 | 0.89 | 20 | 22 |
| **25 minutes** | **10** | **Scoop&Run** | 19.2 | 0.45 | 19 | 20 |
|  |  | **Stay&Play** | 23.2 | 1.30 | 22 | 25 |
|  | **20** | **Scoop&Run** | 19.0 | 0.00 | 19 | 19 |
|  |  | **Stay&Play** | 22.4 | 0.55 | 22 | 23 |
|  | **40** | **Scoop&Run** | 19.2 | 0.45 | 19 | 20 |
|  |  | **Stay&Play** | 20.8 | 0.84 | 20 | 22 |

**Supplementary Table A2:** Results of the linear regression analysis comparing the auto-injector reduction experiment to the control group where this intervention was not performed. Statistically significant P values are marked in bold. There is no significant effect of the auto-injector reduction, nor were there any interactions with other variables.

| Dependent Variable: | Number of preventable deaths | **R-squared:** | 0.766 |
| --- | --- | --- | --- |
| Model: | OLS | **Adj. R-squared:** | 0.764 |
| Method: | Least Squares | **F-statistic:** | 291.5 |
| No. Observations: | 720 | **Prob (F-statistic):** | 1.21e-218 |
| Df Residuals: | 711 | **Log-Likelihood:** | -1448.7 |
| Df Model: | 8 | **AIC:** | 2915. |
| Covariance Type: | nonrobust | **BIC:** | 2957. |

|  | coefficient | Standard error | t-value | p-value | [0.025 | 0.975] |
| --- | --- | --- | --- | --- | --- | --- |
| **Intercept** | 14.7612 | 0.438 | 33.724 | **0.000** | 13.902 | 15.621 |
| **Policy [Stay&Play]** | 3.1222 | 0.136 | 23.002 | **0.000** | 2.856 | 3.389 |
| **Auto-injector reduction [True]** | -0.6798 | 0.612 | -1.112 | 0.267 | -1.880 | 0.521 |
| **AMS team Arrival Time** | 0.2320 | 0.028 | 8.362 | **0.000** | 0.178 | 0.286 |
| **AMS team Arrival Time ***  **Auto-injector reduction [True]** | 0.0540 | 0.039 | 1.376 | 0.169 | -0.023 | 0.131 |
| **Antidote Doses** | -0.1424 | 0.016 | -8.712 | **0.000** | -0.174 | -0.110 |
| **Antidote Doses ***  **Auto-injector reduction [True]** | 0.0268 | 0.023 | 1.160 | 0.247 | -0.019 | 0.072 |
| **AMS team Arrival Time * Antidote Doses** | 0.0058 | 0.001 | 5.503 | **0.000** | 0.004 | 0.008 |
| **AMS team Arrival Time ***  **Antidote Doses ***  **Auto-injector reduction [True]** | -0.0018 | 0.001 | -1.189 | 0.235 | -0.005 | 0.001 |

| Omnibus-test | 5.962 | **Durbin-Watson:** | 1.031 |
| --- | --- | --- | --- |
| Prob.(Omnibus): | 0.051 | **Jarque-Bera (JB):** | 4.874 |
| Skew: | 0.111 | **Prob.(JB):** | 0.0874 |
| Kurtosis: | 2.663 | **Condition Nomber** | 4.89e+03 |

**Supplementary Table A3:** Average last auto-injector application times by AMS team arrival time, and number of auto-injectors (without reduction). SD represents the standard deviation. Both policies have identical values as they only affect the results downstream from the intervention.

| Arrival Time | Auto-injectors | Policy | Mean | SD | Minimum | Maximum |
| --- | --- | --- | --- | --- | --- | --- |
| **9 minutes** | **15** | **Scoop&Run** | 12.89 | 1.15 | 11.48 | 15.18 |
|  |  | **Stay&Play** | 12.89 | 1.15 | 11.48 | 15.18 |
|  | **30** | **Scoop&Run** | 16.86 | 3.38 | 11.48 | 21.52 |
|  |  | **Stay&Play** | 16.86 | 3.39 | 11.48 | 21.48 |
|  | **60** | **Scoop&Run** | 22.35 | 4.81 | 11.48 | 29.44 |
|  |  | **Stay&Play** | 22.33 | 4.79 | 11.48 | 29.37 |
|  | **90** | **Scoop&Run** | 25.89 | 5.91 | 11.48 | 35.27 |
|  |  | **Stay&Play** | 25.87 | 5.89 | 11.48 | 35.38 |
|  | **Maximum** | **Scoop&Run** | 56.36 | 21.63 | 11.48 | 94.27 |
|  |  | **Stay&Play** | 56.27 | 21.60 | 11.48 | 93.81 |
| **11 minutes** | **15** | **Scoop&Run** | 14.45 | 0.55 | 13.45 | 15.30 |
|  |  | **Stay&Play** | 14.45 | 0.55 | 13.45 | 15.30 |
|  | **30** | **Scoop&Run** | 17.51 | 2.73 | 13.45 | 21.85 |
|  |  | **Stay&Play** | 17.51 | 2.74 | 13.45 | 21.95 |
|  | **60** | **Scoop&Run** | 22.54 | 4.38 | 13.45 | 29.24 |
|  |  | **Stay&Play** | 22.55 | 4.38 | 13.45 | 29.20 |
|  | **90** | **Scoop&Run** | 26.07 | 5.64 | 13.45 | 35.20 |
|  |  | **Stay&Play** | 26.06 | 5.61 | 13.45 | 35.39 |
|  | **Maximum** | **Scoop&Run** | 56.34 | 21.57 | 13.45 | 94.10 |
|  |  | **Stay&Play** | 56.30 | 21.57 | 13.45 | 94.01 |
| **13 minutes** | **15** | **Scoop&Run** | 20.17 | 1.97 | 15.46 | 25.07 |
|  |  | **Stay&Play** | 20.17 | 1.98 | 15.46 | 25.10 |
|  | **30** | **Scoop&Run** | 22.28 | 2.76 | 15.46 | 27.74 |
|  |  | **Stay&Play** | 22.27 | 2.76 | 15.46 | 27.64 |
|  | **60** | **Scoop&Run** | 25.72 | 4.18 | 15.46 | 34.19 |
|  |  | **Stay&Play** | 25.72 | 4.17 | 15.46 | 34.02 |
|  | **90** | **Scoop&Run** | 28.80 | 5.63 | 15.46 | 39.69 |
|  |  | **Stay&Play** | 28.79 | 5.61 | 15.46 | 39.54 |
|  | **Maximum** | **Scoop&Run** | 56.62 | 20.97 | 15.46 | 93.58 |
|  |  | **Stay&Play** | 56.72 | 21.03 | 15.46 | 93.70 |
| **15 minutes** | **15** | **Scoop&Run** | 20.43 | 1.67 | 17.23 | 24.97 |
|  |  | **Stay&Play** | 20.44 | 1.69 | 17.23 | 25.04 |
|  | **30** | **Scoop&Run** | 22.55 | 2.64 | 17.23 | 27.57 |
|  |  | **Stay&Play** | 22.55 | 2.65 | 17.23 | 27.62 |
|  | **60** | **Scoop&Run** | 25.95 | 4.11 | 17.23 | 33.91 |
|  |  | **Stay&Play** | 25.99 | 4.15 | 17.23 | 34.12 |
|  | **90** | **Scoop&Run** | 29.03 | 5.62 | 17.23 | 39.52 |
|  |  | **Stay&Play** | 29.07 | 5.64 | 17.23 | 39.89 |
|  | **Maximum** | **Scoop&Run** | 56.69 | 20.93 | 17.23 | 93.32 |
|  |  | **Stay&Play** | 56.73 | 20.94 | 17.23 | 93.48 |
