# Supplementary material for: Impact of antidote quantity, timing and prehospital strategies in nerve agent mass casualty events: a simulation study
Source: Front Public Health. 2025 Aug 26;13:1640554. doi: 10.3389/fpubh.2025.1640554 (PMC12417396; doi:10.3389/fpubh.2025.1640554)
Supplement: Supplementary file 2 [file Supplementary_file_1.docx]

**Strengthening the Reporting of Empirical Simulation Studies (STRESS)**

**Discrete-event simulation guidelines STRESS-DES**

| **Section/Subsection** | **Item** | **Recommendation** | |
| --- | --- | --- | --- |
| 1. **Objectives** |  |  | |
| Purpose of the model | 1.1 | Explain the background and objectives for the model.  ***  The SIMEDIS simulation model:   1. Incorporates the geospatial location of the disaster site, the Casualty Collection Point (CCP), Forward Medical Post (FMP), hospitals, the Advanced Medical Stabilization (AMS) team base, firefighter bases, Mobile Medical Teams (MMTs), ambulances and victims for a given region and calculates routes and transfers times between all these locations. 2. Models the health state of every victim over time from injury to death or hospital arrival (SimedisScore, SS). It uses a Gompertz model based on 5 clinical parameters (Glasgow Coma Scale, Respiratory Rate, Oxygen Saturation, Heart Rate and Blood Pressure), as well as the effects of treatment and antidote application on this health state 3. Implements the Belgian National Contingency Plan, using actual hospital surge capacity and response times, as well as two evacuation policies (Stay&Play and Scoop&Run) 4. Tracks the number of antidote doses and auto-injectors 5. Incorporates stochastic variation on transport/drive times and treatment times to reflect uncertainty and variability inherent to the disaster context. 6. Terminates when all victims are dead or accounted for either at the hospital, at the outpatient clinic (OPC) or at home.   *** | |
| Model Outputs | 1.2 | Define all quantitative performance measures that are reported, using equations where necessary. Specify how and when they are calculated during the model run along with how any measures of error such as confidence intervals are calculated.  ***  The outputs of the model are the victim’s location, treatments over time and SimedisScore value, as well as the locations over time of every ambulance and MMT. An output is generated every time the victim enters a queue or has physical contact with a healthcare worker, who can determine if the victim has died.  The 95% confidence interval was calculated over 5 replications per parameter combination.  *** | |
| Experimentation Aims | 1.3 | If the model has been used for experimentation, state the objectives that it was used to investigate.     1. Scenario based analysis – Provide a name and description for each scenario, providing a rationale for the choice of scenarios and ensure that item 2.3 (below) is completed. 2. Design of experiments – Provide details of the overall design of the experiments with reference to performance measures and their parameters (provide further details in *data* below). 3. Simulation Optimisation – (if appropriate) Provide full details of what is to be optimised, the parameters that were included and the algorithm(s) that was be used. Where possible provide a citation of the algorithm(s).   ***  The objective of this model is to explore operational decisions critical in responding to nerve agent MCIs. The experimentation was divided into four key parts:   1. **Primary Experiment – AMS Arrival Time, Antidote Availability, and Evacuation Policy:**   Objective: Assess how AMS team arrival time, antidote dose quantity, and the choice of hospital transport policies influence overall number of preventable deaths.  Design: Full factorial design varying: AMS arrival times (5–25 minutes in 2-minute intervals), Antidote doses 0, 20, 40) and evacuation policies (Scoop&Run vs. Stay&Play)   1. **Antidote Number Analysis – Focused Exploration of Dose-Response Curve:**   Objective: Examine the sigmoid mortality effect from the primary experiment and determine the marginal benefit of increasing antidote quantities.  Design: Vary antidote doses from 0 up to the theoretical maximum (415 antidote doses and 517 auto-injectors), with fixed AMS arrival times in critical time ranges (e.g., 9–13 minutes).   1. **Bottleneck Analysis – Transport and Resource Constraints in Stay&Play Policy:**   Objective: Identify the impact of transport and FMP resource constraints on prehospital mortality.  Design: Replicate primary scenarios but increase MMTs, ambulance numbers, and FMP staffing to measure mortality improvements.   1. **Auto-injector Reduction Sensitivity – From 3 to 1 Injector per Severe Victim:**   Objective: Evaluate the effect of a possible operation decision taken by the DIR-MED to reduce the antidote dose per victim from 3 auto-injectors to 1 auto-injector.  Design: Maintain original settings but change the per-victim dose assumption 10 minutes after DIR-MED arrival and analyse the impact on preventable deaths.  *** | |
| 1. **Logic** |  |  | |
| Base model overview diagram | 2.1 | Describe the base model using appropriate diagrams and description. This could include one or more process flow, activity cycle or equivalent diagrams sufficient to describe the model to readers. Avoid complicated diagrams in the main text. The goal is to describe the breadth and depth of the model with respect to the system being studied.  ***  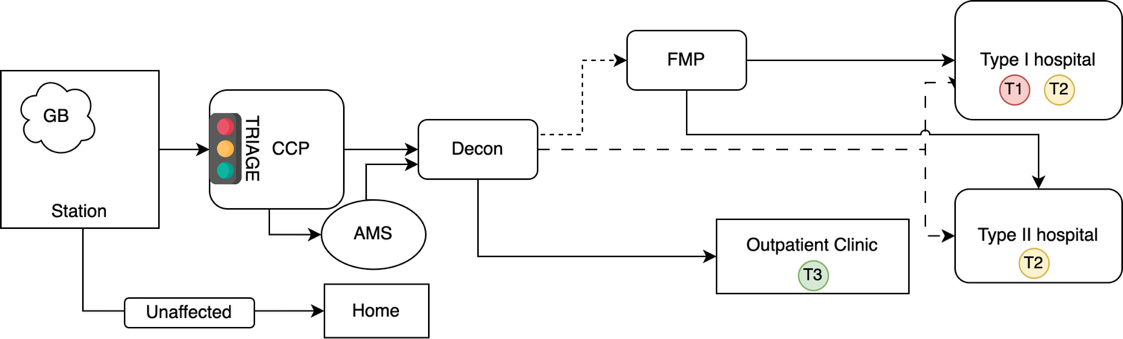  **Figure 1: Schematic representation of the simulator design.** The GB cloud represents the sarin cloud. T1 represents the ‘immediate’ triage category, T2 represents the ‘delayed’ triage category and T3 represents the ‘minimal’ triage category. Decon represents the decontamination station and AMS represents the Advanced Medical Stabilization (AMS) team. After decontamination, T3 victims are sent to outpatient clinics. T1 and T2 victims are sent either to hospitals (coarse dashed line) in the Scoop&Run Policy or are transported to the Forward Medical Post (FMP, fine dashed line) for further stabilization in the Stay&Play policy, after which they also are transported to the hospital.  *** | |
| Base model logic | 2.2 | Give details of the base model logic. Give additional model logic details sufficient to communicate to the reader how the model works.  ***  Each victim enters the simulation at the subway station and is then processed through a discrete chain: self-evacuation or HAZMAT-assisted evacuation → triage (with limited lifesaving interventions) → AMS stabilization (limited to severe cases) → decontamination → transport.  In the Stay&Play policy, victims are routed to the FMP after decontamination. At FMP arrival, all victims are re-triaged. Depending on their triage category and updated health status, victims may receive further treatment at the FMP. This treatment increases survival time (56 minutes for T1, 78 minutes for T2). After FMP treatment, victims enter a queue for hospital transport. During transport, additional treatment may also be provided (26 minutes survival gain for T1, 40 minutes for T2).  Throughout the simulation, victims undergo health state evaluations whenever they encounter a healthcare worker. These encounter points include triage, AMS stabilization, decontamination, FMP arrival, FMP treatment, during hospital selection and during transport. These checks assess whether a victim's survival time has expired and determine if they are declared dead at that point.  *** | |
| Scenario logic | 2.3 | Give details of the logical difference between the base case model and scenarios (if any). This could be incorporated as text or where differences are substantial could be incorporated in the same manner as 2.2.  ***  Each scenario modifies one or more aspects of the base model logic to explore key response decisions:  Primary Experiment: Scenarios vary the AMS team's arrival time, the quantity of available antidotes, and the choice between Scoop&Run and Stay&Play evacuation strategies. The core logic of treatment, triage, and transport remains the same, but the timing and availability of resources are altered.  Antidote Number Analysis: These scenarios isolate the effect of antidote availability by systematically varying the number of antidote doses, while keeping other parameters (AMS arrival time and evacuation strategy) fixed. The base logic is unchanged except for modified stock levels of antidotes.  Bottleneck Analysis: These scenarios quadruple ambulance transport capacity, double MMT availability, and double the FMP minimum staffing to relieve transportation and FMP treatment bottlenecks and to verify the effect observed in the previous scenarios can be traced back to the AMS arrival time and antidote availability.  Auto-injector Reduction Sensitivity: These scenarios simulate the impact of reducing per-victim auto-injector use 10 minutes after DIR-MED arrival. This reduces the number of applied auto-injectors from 3 to 1 auto-injector for severe cases and decreases efficacy.  *** | |
| Algorithms | 2.4 | Provide further detail on any algorithms in the model that (for example) mimic complex or manual processes in the real world (i.e. scheduling of arrivals/appointments/operations/maintenance, operation of a conveyor system, machine breakdowns, etc.). Sufficient detail should be included (or referred to in other published work) for the algorithms to be reproducible. Pseudo-code may be used to describe an algorithm.  ***   - Survival tracking: Each time a victim interacts with a healthcare provider (triage, AMS, FMP, transport), their SimedisScore is recalculated. If the SimedisScore is < 0.001, the victim is marked as deceased. This cut-off point was chosen to merge the asymptotic Gompertz function with the discrete death state according to the expected survival times. - Treatment logic: Treatment effects are modelled as deterministic survival time extensions depending on intervention type and injury severity. Time durations for treatments are sampled from distributions to incorporate stochasticity. - DIR-MED-driven resource reassignment: A rule-based scheduling algorithm controls the DIR-MED's dynamic assignment of medical teams and ambulances throughout the prehospital phase. - Hospital selection: Hospitals are assigned based on the victim’s injury needs (e.g. level 1 vs level 2 hospital, major trauma centre, neurosurgery, cardiothoracic surgery) using the principle of filling the closest available hospital. - Transport supervision: T1 victims are either supervised by a doctor or a nurse, while T2 victims are supervised by Emergency Technicians (EMTs) only   *** | |
| Components | 2.5 | 2.5.1 Entities | Give details of all entities within the simulation including a description of their role in the model and a description of all their attributes.  ***  There are 986 victims, each with a uniquely determined injuries and/or chemical injury profiles (IP) and evolving health state based on those injuries tracked via SimedisScore. The SimedisScore determines victim mobility (i.e. > 17) and triage category  *** |
|  |  | 2.5.2 Activities | Describe the activities that entities engage in within the model. Provide details of entity routing into and out of the activity.  ***   - Evacuation from the subway station to CCP, either self-evacuation or with assistance from the HAZMAT team - Triage at the CCP - AMS intervention (stabilization and antidote administration) at the CCP - Decontamination at the CCP - FMP treatment and re-triage (Stay&Play only) - Hospital transport (outside the CCP or at the FMP - In-transit treatment (by the transport supervisor)   Each activity includes time delays and resource constraints and affects the victim's survival probability.  *** |
|  |  | 2.5.3 Resources | List all the resources included within the model and which activities make use of them.  ***   1. Personnel:  - Emergency physicians: 4 of them are used by the triage activity, 5 of them are used for FMP staffing (or 10 in the ‘no bottleneck’ scenario), the others are used for hospital transport. - Emergency nurses: same as emergency physicians - DIR-MED: takes operational decisions. - AMS-Team: performs advanced medical stabilisation. - HAZMAT-team: consisting of 2 firefighters in PPE who perform the search and rescue of 1 incapacitated victim in the disaster site at the time. - Decontamination tracks: every track has a disrobing spot, decontamination spot and a re-robing spot available which is modelled as a resource.  1. Equipment:  - Antidote doses and auto-injectors: 1 dose contains all treatment materials needed for 1 victim and 3 auto-injectors for severely intoxicated victims and 1 auto-injector for the other intoxicated victims  1. Vehicles:  - Ambulances (includes EMTs) are used for transport from CCP to FMP (5 ambulances) or for hospital transport. When all victims have reached the FMP the 5 ambulances are added to the hospital transport pool. - MMT: transport 1 emergency physician and 1 emergency nurse to the disaster site.  1. Hospitals beds  - Hospitals have specific levels of care (pediatric, trauma center, neurosurgergy, cardiothoracic surgery…) available and treatment capacity available (number of beds per triage category)   *** |
|  |  | 2.5.4 Queues | Give details of the assumed queuing discipline used in the model (e.g. First in First Out, Last in First Out, prioritization, etc.). Where one or more queues have a different discipline from the rest, provide a list of queues, indicating the queuing discipline used for each. If reneging, balking or jockeying occur, etc., provide details of the rules. Detail any delays or capacity constraints on the queues.  ***  All queues are First In First Out (FIFO) in the case of identical priorities.  The Hazmat Team and Triage queue priority is based on the victim’s pretriage category, where lower numbers have the highest priority. This pretriage is based on the injury severity and the intoxication level. The exact algorithm is detailed in point 5.3 below.  Victims in decontamination, transport and FMP treatment queues are prioritized using a priority score based on the triage category derived from the SALT triage method, further subdivided by SimedisScore detailed below with the same FIFO principle. Transport and decontamination priority is kept up to date by a specific function that traverses the queue, taking 30 seconds per person in the queue to update their priority score.  *** |
|  |  | 2.5.5 Entry/Exit Points | Give details of the model boundaries i.e. all arrival and exit points of entities. Detail the arrival mechanism (e.g. ‘thinning’ to mimic a non-homogenous Poisson process or balking)  ***  Victims enter the simulation at the subway station (exposure point). They exit after dying, arriving at a hospital ED (T1/T2) or after starting transport to the OPCs (T3). Geographic movement and resource routing are calculated using OpenStreetMap data and Dijkstra’s algorithm for shortest paths.  *** |
| 1. **Data** |  |  | |
| Data sources | 3.1 | List and detail all data sources. Sources may include:   - Interviews with stakeholders, - Samples of routinely collected data, - Prospectively collected samples for the purpose of the simulation study, - Public domain data published in either academic or organisational literature. Provide, where possible, the link and DOI to the data or reference to published literature.   All data source descriptions should include details of the sample size, sample date ranges and use within the study.  ***   - Interviews with stakeholders: members of the current AMS team (CBRNe MMT of Military Hospital Queen Astrid), CBRNe expert of the Brussels firefighters, disaster medicine experts at the Vrije Universiteit Brussel and CBRNe experts of the Belgian Armed Forces were used to model the operational dynamics of the HazMat team, decontamination and AMS treatment. - Samples of routinely collected data from the national 112 call center (determine alert time of resources) and an internal exercise of the Belgian AMS team (decontamination and disrobing speed, AMS treatment speed). - Prospectively collected samples for the purpose of the simulation study: special measurements were performed at the metro station, in combination with a computational fluid dynamics model (1,2). - Public domain data published in either academic or organizational literature: victim model, injury model and treatment times (3–6).   *** | |
| Pre-processing | 3.2 | Provide details of any data manipulation that has taken place before its use in the simulation, e.g. interpolation to account for missing data or the removal of outliers.  ***  The simulation integrates data from multiple sources, including chemical dispersion models, a pharmacokinetic/pharmacodynamic model, military and civilian injury models, and expert-derived clinical parameters. These datasets were harmonized into a unified victim profile structure, combining trauma and chemical exposure severity. A derived health severity index (SimedisScore) was computed from vital signs and intoxication levels.  No interpolation or outlier removal was required due to the simulated nature of the data. All pre-processing involved expert-based assignment and rule-based combination of discrete injury and exposure profiles. The resulting victim set (986 individuals) was consistently applied across all simulation runs.  *** | |
| Input parameters | 3.3 | List all input variables in the model. Provide a description of their use and include parameter values. For stochastic inputs provide details of any continuous, discrete or empirical distributions used along with all associated parameters. Give details of all time dependent parameters and correlation.  Clearly state:   - Base case data - Data use in experimentation, where different from the base case. - Where optimization or design of experiments has been used, state the range of values that parameters can take.   Where theoretical distributions are used, state how these were selected and prioritised above other candidate distributions.  ***  Key input parameters and their characteristics include:   1. Resource locations: MMT stations, firefighter bases, hospitals, disaster site, CCP, FMP and Transport zone locations. All locations are encoded by the latitude and longitude coordinates, either exact or the point of entrance at street level. Resource activation times 2. Injury Distribution: Victim profiles (chemical, traumatic, combined) derived from validated modeling frameworks. 3. Hospital Capacity: Defined by the real-life surge and treatment capacity. 4. AMS Arrival Time: Varied from 5 to 25 minutes in 2-minute increments (scenario dependent). 5. Antidote Doses: 0 to 517 available injectors (scenarios include intermediate values and theoretical maximum). 6. Transport Times and Routing: Derived from geographic data (OpenStreetMap) and routed using Dijkstra's algorithm with. 7. Treatment Durations and Activities: Drawn from expert input and literature-informed distributions:    - Incident Recognition Time: Constant 2.5 minutes    - Walking Speed: Normal distribution, 1.3 m/s mean, ¬±20% (3œÉ capped)    - Triage Duration: Truncated normal; 30s (incapacitated), 5s (mobile victims    - Decontamination Time:      - Mobile: Mean 3.66 min ¬±25%      - Incapacitated: Mean 5 min ¬±25%    - AMS Treatment Time: T1: 4 min, T2: 2 min, T3: 2 min (triangular, ¬±20%)    - Chemical Treatment: T1: 10 min, T2: 5 min, T3: 2 min (triangular, ¬±20%)    - Traumatic Treatment: T1: 15 min, T2: 10 min, T3: 5 min (triangular, ¬±20%)    - Ambulance Loading/Unloading:      - Loading: Mean 2 min ¬±20%      - Unloading: Mean 14 min ¬±20%    - Surge Capacity Refresh: Every 2 hours    - FMP Readiness Time: Constant 15 minutes    - Decontamination Readiness: Available 15 minutes post-incident. 8. Resource counts: Ambulance Count: 29; MMT Count: 14; Triage MMTs: 4; FMP Staff: 5 doctors, 5 nurses   Time-dependent behavior (e.g., resource availability, hospital capacity refresh) is modeled using rule-based schedules. All probability distributions (where used) are based on expert judgment or literature-informed estimates, as empirical data for CBRN mass casualty contexts is lacking.  *** | |
| Assumptions | 3.4 | Where data or knowledge of the real system is unavailable what assumptions are included in the model? This might include parameter values, distributions or routing logic within the model.  ***  Where real-world data was unavailable or incomplete, the following assumptions were used:   1. Clinical Response Consistency: All responders perform tasks without error or delay beyond the modelled timelines (idealized execution). 2. Fixed Treatment Effects: Interventions provide consistent, predefined extensions in survival time. 3. No Bystander or Walk-in Behavior: Only modeled victims from the scenario are considered (e.g., no self-evacuating walk-ins to hospitals). 4. Simplified Dry Decontamination: Modelled as a uniform process with time delays, not differentiated by contamination severity or victim cooperativeness. 5. Adequate Resource availability and/or reusability: Medical personnel and ambulances return to the site after transport, without delay from restocking or debriefing. 6. No Expectant Category: All victims are considered treatable under optimal conditions; none are triaged as beyond salvage (T4). 7. Uniform Application of Triage Protocols: SALT triage applied identically across all MMTs without variability.   *** | |
| 1. **Experimentation** |  |  | |
| Initialisation | 4.1 | Report if the system modelled is terminating or non-terminating. State if a warm-up period has been used, its length and the analysis method used to select it. For terminating systems state the stopping condition.  State what if any initial model conditions have been included, e.g., pre-loaded queues and activities. Report whether initialization of these variables is deterministic or stochastic.  ***  The model is terminating; there is no warm-up period. The initial conditions are deterministic. All resources start at their respective base. Victims are initialized based on a social force subway evacuation model in combination with a gaussian puff sarin release model, based on the subway stations geometry.  *** | |
| Run length | 4.2 | Detail the run length of the simulation model and time units.  ***  Model initialization including map loading takes +- 60 seconds. 1 simulation replication takes between 6 and 8 seconds.  *** | |
| Estimation approach | 4.3 | State the method used to account for the stochasticity: For example, two common methods are multiple replications or batch means. Where multiple replications have been used, state the number of replications and for batch means, indicate the batch length and whether the batch means procedure is standard, spaced or overlapping. For both procedures provide a justification for the methods used and the number of replications/size of batches.  ***  Stochasticity in the simulation was addressed using the multiple replications method, with 5 independent replications conducted for each parameter combination. For each replication, a random seed was generated and fixed per parameter combination.  The choice of 5 replications is a balance between computational feasibility and statistical robustness, determined through preliminary tests showing that key output metrics (e.g., evacuation and response times) exhibited acceptable levels of variability and convergence. Batch means were not used.  *** | |
| 1. **Implementation** |  |  | |
| Software or programming language | 5.1 | State the operating system and version and build number.  State the name, version and build number of commercial or open source DES software that the model is implemented in.  State the name and version of general-purpose programming languages used (e.g. Python 3.5).  Where frameworks and libraries have been used provide all details including version numbers.  ***  Operating System: MacOS Sonoma 14.5  DES Software: SimJulia 0.8.2 (recently renamed to ConcurrentSim). using Julia 1.8  Mapping was done via OpenStreetMapX 0.4 Julia Package.  *** | |
| Random sampling | 5.2 | State the algorithm used to generate random samples in the software/programming language used e.g. Mersenne Twister.  If common random numbers are used, state how seeds (or random number streams) are distributed among sampling processes.  ***  Julia's default random number generator was used: (Mersenne Twister).  *** | |
| Model execution | 5.3 | State the event processing mechanism used e.g. three phase, event, activity, process interaction.  *Note that in some commercial software the event processing mechanism may not be published. In these cases authors should adhere to item 5.1 software recommendations.*  State all priority rules included if entities/activities compete for resources.  If the model is parallel, distributed and/or use grid or cloud computing, etc., state and preferably reference the technology used. For parallel and distributed simulations, the time management algorithms used. If the HLA is used then state the version of the standard, which run-time infrastructure (and version), and any supporting documents (FOMs, etc.)  ***  The processing mechanism is process interaction with scheduled events. All queues function on a FIFO principle with an optional integer based priority. When a priority score is used, the lowest priority score gets picked first.  Priority rules for treatment, decontamination and transport queues are based on triage category and SimedisScore. The priority score used calculated as *triage_category * 100 + trunc(SimedisScore)*. The lowest priority score has the highest priority. Triage categories range from 1 to 3, with 1 being the most urgent and 3 being the least urgent. In the decontamination queue this priority score is updated by a function that continuously traverses the queue, taking 30 seconds per patient  The triage queue and evacuation queue have priorities modeled on the pretriage category. The pretriage algorithm determines the triage level based on the ISS value and IP level. The rules are as follows:   - If the ISS value is 25 or higher, the triage level is 1. - If the ISS value is between 5 and 24 (inclusive), the triage level is 2. - If the ISS value is either 3 or 4, the triage level is 3. - If the ISS value is less than 1, the triage level is 5. - If the IP level is 5 or higher, the triage level is 1. - If the IP level is 2 or lower, the triage level is 3. - If the IP level is 7 (which corresponds to a dose of 0.0), the triage level is 5. - If the IP level is between 3 and 4 (inclusive), the triage level is 2. - The final triage level is the minimum of the triage levels determined by the ISS value and IP level.   The model is not parallelized nor distributed.  *** | |
| System Specification | 5.4 | State the model run time and specification of hardware used. This is particularly important for large scale models that require substantial computing power. For parallel, distributed and/or use grid or cloud computing, etc. state the details of all systems used in the implementation (processors, network, etc.)  ***  The model was run on a 2020 iMac with a 3,1 GHz 6-Core Intel Core i5 and 24GB DDR4 Ram.  *** | |
| 1. **Code Access** |  |  | |
| Computer Model Sharing Statement | 6.1 | Describe how someone could obtain the model described in the paper, the simulation software and any other associated software (or hardware) needed to reproduce the results. Provide, where possible, the link and DOIs to these.  ***  All software packages needed to reproduce the results is open source and is available on their respective public repositories.  The SIMEDIS software is property of the Vrije Universiteit Brussel and the Royal Military Academy. It is not available for distribution. Interested parties may contact the corresponding author (Ruben De Rouck) for specific questions on reproducing the results.  *** | |
| 1. **References** |  |  | |
|  |  | ***  1. Faugier L, Marinus BG, Bosschaerts W, Laboureur D, Limam K. CFD model to assess parameters influencing piston wind in a subway tunnel and station. *J Phys: Conf Ser* (2021) 2042:012076. doi: 10.1088/1742-6596/2042/1/012076  2. Faugier L, Marinus BG, Bosschaerts W, Laboureur D, Limam K. CFD model for airflow in a subway station compared to on-site measurements: The challenges of as-built environment. *Tunnelling and Underground Space Technology* (2023) 140:105248. doi: 10.1016/j.tust.2023.105248  3. Udayasiri R, Knott J, McD Taylor D, Papson J, Leow F, Hassan FA. Emergency department staff can effectively resuscitate in level C personal protective equipment. *Emerg Med Australas* (2007) 19:113–121. doi: 10.1111/j.1742-6723.2007.00918.x  4. Blomme L, Pauwels S, De Rouck R, Hubloue I. Assessment of the decontamination procedure for self-referrals of a chemical mass casualty disaster, through a simulation exercise at the emergency department of a University Hospital in Brussels (UZ Brussel). (2020)  5. Benhassine M, Van Utterbeeck F, De Rouck R, Debacker M, Dhondt E, Hubloue I. Simulating the Evacuation of a Subway Station after a Sarin Release. *Proceedings of the 36th European Simulation Conference*. Porto, Portugal: EUROSIS-ETI (2022). p. 271–7  6. De Rouck R, Benhassine M, Debacker M, Dugauquier C, Dhondt E, Van Utterbeeck F, Hubloue I. Creating realistic nerve agent victim profiles for computer simulation of medical CBRN disaster response. *Front Public Health* (2023) 11:1167706. doi: 10.3389/fpubh.2023.1167706  *** | |
